# Cholinergic Abnormalities, Endosomal Alterations and Up-Regulation of Nerve Growth Factor Signaling in Niemann-Pick Type C Disease

**DOI:** 10.1186/1750-1326-7-11

**Published:** 2012-03-29

**Authors:** Carolina Cabeza, Alicia Figueroa, Oscar M Lazo, Carolina Galleguillos, Claudia Pissani, Andrés Klein, Christian Gonzalez-Billault, Nibaldo C Inestrosa, Alejandra R Alvarez, Silvana Zanlungo, Francisca C Bronfman

**Affiliations:** 1 Physiology Department. Millennium Nucleus in Regenerative Biology (MINREB). Faculty of Biological Sciences, Pontificia Universidad Católica de Chile, Alameda 340, Santiago 8320000, Chile; 2 Department of Cellular and Molecular Biology. Faculty of Biological Sciences, Pontificia Universidad Católica de Chile, Alameda 340, Santiago 8320000, Chile; 3 Department of Gastroenterology. Faculty of Medicine, Pontificia Universidad Católica de Chile, Alameda 340, Santiago 8320000, Chile; 4 Center for Aging and Regeneration (CARE), Alameda 340, Santiago 8320000, Chile; 5 Laboratory of Cell and Neuronal Dynamics, Dept. Biology and Institute for Cell Dynamics and Biotechnology (ICBD), Faculty of Sciences, Universidad de Chile, Las Palmeras 3425, Santiago 8320000, Chile

**Keywords:** NGF, Endosomes, Cholesterol, Niemann-Pick type C1, cholinergic system, PC12

## Abstract

**Background:**

Neurotrophins and their receptors regulate several aspects of the developing and mature nervous system, including neuronal morphology and survival. Neurotrophin receptors are active in signaling endosomes, which are organelles that propagate neurotrophin signaling along neuronal processes. Defects in the *Npc1 *gene are associated with the accumulation of cholesterol and lipids in late endosomes and lysosomes, leading to neurodegeneration and Niemann-Pick type C (NPC) disease. The aim of this work was to assess whether the endosomal and lysosomal alterations observed in NPC disease disrupt neurotrophin signaling. As models, we used i) NPC1-deficient mice to evaluate the central cholinergic septo-hippocampal pathway and its response to nerve growth factor (NGF) after axotomy and ii) PC12 cells treated with U18666A, a pharmacological cellular model of NPC, stimulated with NGF.

**Results:**

NPC1-deficient cholinergic cells respond to NGF after axotomy and exhibit increased levels of choline acetyl transferase (ChAT), whose gene is under the control of NGF signaling, compared to wild type cholinergic neurons. This finding was correlated with increased ChAT and phosphorylated Akt in basal forebrain homogenates. In addition, we found that cholinergic neurons from NPC1-deficient mice had disrupted neuronal morphology, suggesting early signs of neurodegeneration. Consistently, PC12 cells treated with U18666A presented a clear NPC cellular phenotype with a prominent endocytic dysfunction that includes an increased size of TrkA-containing endosomes and reduced recycling of the receptor. This result correlates with increased sensitivity to NGF, and, in particular, with up-regulation of the Akt and PLC-γ signaling pathways, increased neurite extension, increased phosphorylation of tau protein and cell death when PC12 cells are differentiated and treated with U18666A.

**Conclusions:**

Our results suggest that the NPC cellular phenotype causes neuronal dysfunction through the abnormal up-regulation of survival pathways, which causes the perturbation of signaling cascades and anomalous phosphorylation of the cytoskeleton.

## Background

Neurotrophins (NGF, BDNF, NT3 and NT4) regulate different aspects of the developing and mature nervous system, including neuronal survival and neuronal morphology. These small proteins exert these effects by binding to members of the Trk family of receptor tyrosine kinases (TrkA, TrkB and TrkC) or to the p75 neurotrophin receptor (p75). Whereas p75 binds all neurotrophins, in addition to other ligands (e.g., proneurotrophins and amyloid peptides), each Trk binds preferentially to its cognate neurotrophin. For example, TrkA, TrkB and TrkC bind NGF, BDNF and NT3, respectively [1-3].

Several neurodegenerative diseases are produced by alterations in molecules related to endocytosis and vesicular trafficking, which are cellular processes that regulate neurotrophin signaling [4,5]. Therefore, one possible target of endosomal abnormalities and trafficking defects is neurotrophin signaling [6,7].

Niemann-Pick type C (NPC) disease is a fatal autosomal recessive disorder resulting from mutations in the *NPC1 *(in 95% of patients) or the *NPC2 *gene (in 5% of patients). The loss of NPC1 or NPC2 function causes the accumulation of cholesterol and glycosphingolipids in the late endocytic pathway. Some evidence has suggested that the accumulation of cholesterol and other lipids inside the cell results in endosomal abnormalities, including alterations in the recycling pathway and alterations in late endosome dynamics, in addition to the down-regulation of neurotrophic signaling [8-17]. Although most mammalian cells are affected by intracellular cholesterol overload, neurodegeneration is the main cause of fatality in patients with NPC disease [8,10].

NPC disease shares several similarities, including late endosomal and lysosomal abnormalities, neurofibrillary tangles and cognitive impairment, with other neurodegenerative disorders, such as Alzheimer's disease (AD) [18,19]. A typical NPC patient will develop cerebellar ataxia and progressive cognitive deterioration, in addition to compromised organ function [8,11,20]. In AD, cognitive impairment is correlated with neurodegeneration of the central cholinergic system. Basal forebrain cholinergic neurons account for most of the cholinergic innervation of the hippocampus and cortical mantle and play a key role in the regulation of synaptic activity and the modulation of memory and attention [21-24]. Derangement of the cholinergic system is one of the pathological hallmarks of AD and contributes to the progressive cognitive deterioration of AD patients [25]. NPC patients also show cognitive decline [20], but no study has examined the possible causes underlying the neuropathological alterations associated with cognitive impairment in this disease [26,27].

The aim of this work was to assess whether the endosomal alterations that are observed in mouse and cellular models of NPC disease disrupt NGF signaling. We used two different models: NPC1-deficient (*NPC1*^-/-^) mice and PC12 cells treated with U18666A (PC12-U18666A cells), a well-known inducer of the NPC phenotype [28-31]. Contrary to our expectations, NGF signaling was up-regulated or conserved in both models of NPC, suggesting that neurodegeneration in NPC may result from the misregulation of kinase cascades triggered by neurotrophins as well as other trophic factors.

## Methods

Goat polyclonal anti-ChAT antibody was obtained from Chemicon (Temecula, CA, USA). Rabbit polyclonal anti-p75, mouse monoclonal anti-PLCγ and rabbit polyclonal anti-TrkA antibodies were obtained from Upstate Biotech (Charlottesville, USA). Mouse monoclonal anti-tau (AT8) and mouse monoclonal anti-Flag antibodies were obtained from Pierce (Rockford, Illinois, USA). Mouse monoclonal anti-neurofilament antibody was obtained from Sigma (St. Louis, Missouri, USA). Biotinylated secondary antibodies against rabbit and mouse IgG and protease-free serum bovine albumin (BSA) were obtained from Jackson ImmunoResearch (West Grove, Pennsylvania, USA). The following were obtained from Molecular Probes (Invitrogen, Maryland, USA): mouse monoclonal antibody against Alexa Fluor 488; Alexa Fluor 488 carboxylic acid, 2,3,*5*,6-tetrafluorophenyl ester (Alexa Fluor 488 5*-*TFP); transferrin conjugated to Alexa Fluor 568; phalloidin Alexa Fluor 546; LysoTracker Red DND-99; Alexa Fluor 488-conjugated donkey anti-rabbit antibody; Alexa Fluor 594-conjugated donkey anti-goat antibody; Alexa Fluor 488-conjugated donkey anti-mouse antibody; and Alexa Fluor 555-conjugated cholera toxin subunit-B (CT-B). NGF was obtained from Alamone Labs (Jerusalem, Israel). ALZET Osmotic pumps (model 1002) were obtained from Alza Corp. (Palo Alto, California, USA). The ABComplex-peroxidase kit and 3-'3-diaminobenzidine were obtained from DakoCytomation (California, USA). Mowiol and phosphatase inhibitor cocktail were obtained from Calbiochem (San Diego, California, USA). Wortmannin was obtained from Sigma-Aldrich (St. Louis, USA). U18666A was obtained from Biomol (Plymouth Meeting, Pennsylvania, USA). Phospho-PLC-γ rabbit polyclonal, phospho-ERK1/2 rabbit polyclonal, ERK1/2 rabbit polyclonal and Akt rabbit polyclonal antibodies were obtained from Cell Signaling (Beverly, Massachusetts, USA). Phospho-Akt rabbit polyclonal antibody was obtained from Santa Cruz Biotechnology (Santa Cruz, California, USA). The protease inhibitor cocktail was obtained from Roche (Hertfordshire, UK). All other reagents were obtained from Sigma (St. Louis, Missouri, USA). The plasmid for Rab7-GFP was a gift from Cecilia Bucci (University of Salento, Lecce, Italy). The plasmid for Rab5-GFP was a gift from Victor Faundez (Emory University, USA). The plasmid for TrkA-Flag was a gift from Francis Lee (Cornell University, NY, USA).

### Animals

BALB/c mice carrying a heterozygous mutation in the *Npc1 *gene were kindly donated by Dr. Peter Pentchev (U.S. National Institutes of Health, Bethesda, MD, USA). The genotypes were determined by polymerase chain reaction (PCR)-based screening as described previously [32]. All animal protocols were approved by the Animal Studies Review Board of our institution.

Mice were anesthetized with a mixture of xylazine (12 mg/kg) and ketamine (80 mg/kg) administered i.p., and 9% lidocaine was applied locally to the ears. Mice were then positioned in a stereotaxic apparatus, and coordinates were calculated based on the Franklin and Paxinos atlas of the mouse brain [33]. After surgery, the animals were injected i.p. with the antibiotics ketoprofen (2.5 mg/kg) and enrofloxacin (5 mg/kg) for four days and maintained under observation and temperature control for one week. For histological preparation of brain tissue, anesthetized mice were transcardially perfused with 40 ml of 0.9% NaCl and 20 ml of 4% paraformaldehyde (PFA) in phosphate buffer. After extraction, the brain was post-fixed overnight in 4% PFA, left in 30% sucrose for 24 hrs, and coronally sectioned (40 μm) on a cryostat.

### Fimbria-fornix transection and intracerebroventricular infusion of NGF

Unilateral axotomy of the septo-hippocampal pathway was induced by aspirative lesion of the fimbria-fornix as previously described [34]. In brief, anesthetized mice were positioned in a stereotaxic apparatus, and a small piece of skull was removed at the stereotaxic coordinates 0.6 mm caudal to the bregma and 0.0-2.0 mm lateral to the midline. After excision of the dura, we performed a syringe aspiration of the dorsal fornix-fimbria with a 5-ml syringe fitted with a 25 G 5/8" needle. We also used the syringe to aspirate part of the cingulate and parietal cortices. Mice were sacrificed 7 days after axotomy.

Artificial cerebrospinal fluid (ACSF; 150 mM NaCl, 1.8 mM CaCl_2_, 1.2 mM MgSO_4_, 2 mM KH_2_PO_4 _and 10 mM glucose, pH 7.4) with or without 20 μg/ml NGF (100 μl per osmotic pump) was infused for 7 days with a brain infusion kit (Alza Corp.) connected to a model 1002 ALZET osmotic pump, as described previously [34]. The cannulae and connector tubes were filled with ACSF or ACSF plus NGF and attached to a filled pump. Using the arm of the stereotaxic apparatus, each cannula was lowered into the brain at the coordinates of the left ventricle (0.2 mm caudal to bregma, 0.8 mm to the midline of the contralateral side and 1.8 mm ventral to the brain surface). The cannula was anchored to the skull with a screw and glued with dental acrylic. Axotomized mice were infused with NGF for 7 days immediately following axotomy.

### AChE enzyme histochemistry

Serial coronal cryostat sections (40 μm) were collected in 0.1 M phosphate buffer (pH 7.4), washed in 65 mM sodium maleate (pH 6.0) and incubated as floating sections for staining for 1 hr at room temperature (RT) in a solution containing 0.05 mg/mL acetylthiocholine iodide, 0.1 mM tetra-isopropyl-pyrophosphatamide, 0.05 mM potassium ferricyanide, 0.3 mM CuSO_4_, 0.5 mM sodium citrate and 65 mM sodium maleate (pH 6.0), as described previously [35].

### Immunohistochemistry of brain sections

Immunohistochemistry was performed as follows: (i) 15 min incubation in 0.03% H_2_O_2 _in 0.1 M Tris-HCl, and 150 mM NaCl, pH 7.4 (TBS) to block endogenous peroxidase; (ii) 30 min incubation at 4°C in 0.4% Triton X-100 in TBS; (iii) 1.5 hr incubation at 4°C in 0.2% Triton X-100, 5% fetal calf serum (FCS) and 5% BSA in TBS; (iv) 24 hr incubation at 4°C with primary antibody in TBS plus 0.2% Triton-X100 and 5% FCS; (v) 1.5 hr incubation at RT with biotin-conjugated secondary antibody; and (vi) 1 hr incubation with peroxidase-conjugated avidin ABC (DakoCytomation), followed by visualization of peroxidase activity with diaminobenzidine (DAB, 1 mg/mL) and 0.01% H_2_O_2 _in TBS. Primary antibodies were used at the following dilutions: anti-ChAT (1:300), anti-p75 (1:500) and anti-neurofilament (1:2000). Sections were immediately rinsed in distilled water, mounted, air-dried, cleared with xylene and mounted in Entellan.

### Immunofluorescence of brain sections

Single or double immunofluorescence was performed in floating brain sections as follows: (i) 15 min incubation in TBS; (ii) 15 min incubation in 0.15 M glycine in TBS; (iii) 15 min incubation in TBS; (iv) 10 min incubation in NaBH_4 _(10 mg/ml) in TBS; (iii) 30 min incubation at 4°C in 0.3% Triton X-100 in TBS; (iv) 1.5 hr incubation at 4°C with 0.2% Triton X-100, 5% FCS, and 2% BSA in TBS; (v) 15 min incubation in TBS; (vi) 24 hr incubation at 4°C with primary antibodies in 0.2% Triton X-100, 5% FCS and 2% BSA in TBS; (vii) 15 min incubation in TBS; and (vii) 1.5 hr incubation at RT with fluorochrome-conjugated 2% BSA in TBS. Sections were immediately rinsed in distilled water and mounted in Mowiol.

NeuroTrace (fluorescent Nissl stain; Molecular Probes) staining was performed by incubating brain sections for 20 min in a 1:200 dilution of NeuroTrace in TBS. Fluoro-Jade C (a specific marker for degenerating neurons; Chemicon) staining was performed after NeuroTrace as follows. Brain sections were rinsed twice in TBS, re-hydrated for 2 min in distilled water and incubated for 10 min in 0.06% potassium permanganate. Finally, brain sections were washed for 2 min in distilled water and incubated for 10 min in 0.0002% Fluoro-Jade C solution in 0.1% acetic acid. Sections were immediately rinsed in distilled water, mounted, air dehydrated, cleared with xylene and mounted in Entellan.

Cholesterol staining with filipin, a polyene macrolide that binds to free cholesterol, was performed in brain sections that were previously labeled for ChAT by immunohistochemistry [36]. Sections were incubated with filipin (50 μg/mL) in TBS for 2 hrs at RT in the dark. Sections were then washed in TBS for 15 min and mounted in Mowiol.

The cholinergic cell count was performed as described previously [35]. Septal cholinergic neurons were defined using anatomical landmarks in accordance with the mouse brain atlas [33]. The anterior commissure and anterior and lateral ventricles defined the ventral border of the medial septum (MS). The meeting of the body of the corpus callosum at the midline marked the anterior boundary of the MS, and the midline crossing of the anterior commissure and the appearance of the fornix marked the posterior boundary. Four 40-μm-thick coronal sections per mouse were taken through the complete MS with a 160-μm interval between each section to avoid counting the same cells twice (coordinates from BREGMA: 1.10 to 0.5 mm). ChAT-immunopositive cells were counted on images digitized with an Olympus BX51 (Tokyo, Japan) microscope. The criterion for identifying ChAT-immunopositive cells was the appearance of a clear nuclear shape or, in cases when staining was too dark, clear neuronal morphology.

### Cell culture

PC12 cells were grown in DMEM containing L-glutamine and high glucose and supplemented with 6% horse serum, 6% fetal calf serum and 100 U/ml penicillin-streptomycin. For differentiation assays, PC12 cells were seeded at a density of 10-15,000 cells per square centimeter on poly-D-lysine-coated 16-mm coverslips and maintained for 24, 48 or 72 hrs in the presence of NGF (5 ng/ml) in DMEM/glutamine/high glucose supplemented with 1 mg/ml BSA. Cells were considered differentiated when they had at least one neurite that was twice the diameter of the cell body.

To reproduce the cellular phenotype of NPC disease, PC12 cells were treated with different concentrations (0.5-2 mg/ml) of the drug U18666A, which causes the accumulation of cholesterol in the late endosomes and lysosomes [31].

For filipin staining of PC12 cells, cells were fixed for 10 min with cold paraformaldehyde (4%) in PBS, rinsed twice with cold PBS, incubated for 10 min in glycine (150 mM, pH 7.4) and rinsed three times with PBS. After fixation, the cells were permeabilized for 30 min with saponin (0.2%) and BSA (3%) in PBS, incubated for 2 hrs with filipin at RT (90 μg/ml in PBS), rinsed with PBS and mounted in Mowiol.

### PC12 cell labeling

For immunostaining of PC12 cells with the lysosomal marker LAMP2, cells were fixed and permeabilized as described above, incubated for 1 hr at RT with a rabbit polyclonal antibody against LAMP2 (1:1000) in PBS containing saponin (0.05%) and BSA (3%) and rinsed three times with PBS. PC12 cells were subsequently incubated in the same buffer containing Alexa Fluor 488-conjugated donkey polyclonal antibody against rabbit IgGs (1:400) for 2 hrs in the presence of filipin, then rinsed and mounted in Mowiol.

For Tau immunostaining of differentiated PC12 cells, cells were seeded as described above for the differentiation assays and maintained for 24 hrs with NGF (5 ng/ml) in DMEM/glutamine/high glucose supplemented with 1 mg/ml BSA. Then, the cells were treated for another 48 hrs with or without U18666A (2 μg/ml) in the same buffer. Tau immunostaining of PC12 cells was performed essentially as described in [37]. Briefly, after treatments, PC12 cells were rinsed with warmed extraction buffer (EB) containing MgCl_2 _(1 mM), EGTA (1 mM), GTP (1 mM), glycerol (30%) and PIPES buffer (70 mM), pH 6.9; incubated for 30 seconds with EB containing saponin (0.2%); and rinsed again with EB. PC12 cells were then fixed in EB containing paraformaldehyde (2%) and glutaraldehyde (0.2%) for 1 hr at room temperature. This procedure was followed by a rinse with PBS and cell permeabilization in PBS supplemented with Triton X-100 (0.2%) for 2 min. Cells were blocked by treatment with PBS containing BSA (4%). PC12 cells were then incubated with a primary monoclonal antibody against TAU5 (1:100) or AT8 (1:200) overnight at 4°C. PC12 cells were then rinsed and incubated for 90 min at RT with donkey anti-mouse Alexa 488 (TAU5) or Alexa 594 (AT8) antibody, rinsed and mounted in Mowiol.

### Transfection of PC12 cells with rabs and imaging

PC12 cells were transfected with Lipofectamine-2000 and plasmids containing the cDNA of Rab5-EGFP or Rab7-EGFP according to the manufacturer's instructions. After 24 hrs, PC12 cells were treated for an additional 24 hrs with or without U18666A (2 μg/ml), fixed and processed for filipin staining as described above.

PC12 cells were transfected with the Rab7-EGFP plasmid and, 24 hrs later, treated with U18666A (2 μg/ml) for 24 hrs in the presence of NGF (to increase cell soma). After treatment, PC12 cells were washed three times with PBS, and the coverslip was inverted in 100 μl of warmed incubation media in a silicon rubber chamber on a microscope slide. Rab7-labeled endosome dynamics were imaged in living cells.

For time-lapse visualization of Rab7-positive endosomes in PC12 cells, Rab7-labeled endosome dynamics were imaged in vivo and digitized by capturing one frame every 0.942 sec for approximately 2 min (a total of 250 frames) with a FluoView software station. Images were captured with an Olympus FluoView 1000 with a filter-type detector confocal microscope mounted on an inverted IX81 motorized microscope. Time-lapse experiments were performed with the 488-nm laser line of a multi-argon laser at 20% transmittance with an UPLS APO100xO 1.4NA oil immersion objective. Images were captured with Olympus Software FV10-SW with a pixel resolution of 512 × 512.

### Endocytosis of GM1 and endogenous TrkA in PC12 cells

For endocytosis of GM1 and immunoendocytosis of TrkA, PC12 cells were seeded to a final confluence of 50%, and U18666A (2 μg/ml) was added after 12 hrs. After 24 hrs, PC12 cells were serum-starved for 90 min in 1 mg/ml BSA in DMEM/glutamine/high glucose/HEPES without phenol red (incubation media) and washed with cold PBS. To visualize GM1 accumulation in endosomes, PC12 cells were incubated for 30 min at 37°C with Alexa Fluor 555-conjugated cholera toxin subunit-B (CT-B) (20 μg/ml), washed, fixed and mounted in Mowiol. To visualize the internalization of endogenous TrkA into endosomes, PC12 cells were incubated with 10 μg/ml rabbit TrkA (Upstate) polyclonal antibody in incubation media for 1 hr at 4°C. Then, PC12 cells were incubated for an additional hour with a donkey anti-rabbit Alexa Fluor 488-conjugated antibody (8 μg/ml), washed, and incubated for 2 hrs at 37°C. Cells were then washed, fixed and mounted in Mowiol.

### Internalization and recycling of TrkA-flag in PC12 cells

PC12 cells were seeded to a final confluence of 50%. After 12 hrs, U18666A was added (2 μg/ml) for an additional 24 hrs. Lipofectamine-2000 was then used according to the manufacturer's instructions to transfect PC12 cells with a plasmid expressing TrkA with a Flag epitope tag at its amino terminus (TrkA-Flag). After transfection, U18666A (2 μg/ml) was added for an additional 24 hrs. Afterward, PC12 cells were serum-starved for 90 min in incubation media, washed with cold PBS, treated for 15 min at 37°C with Alexa Fluor 488-labeled monoclonal anti-Flag antibody (8.8 μg/ml), washed and incubated for an additional 1 hr and 45 min at 37°C in the presence of NGF (50 ng/ml). For recycling experiments, the anti-Flag Alexa Fluor 488 antibody was allowed to internalize in the presence of NGF for 45 min. Then, PC12 cells were washed with ice-cold EDTA (1 mM) in PBS three times for 3 min (to remove the anti-Flag antibody, as described by [38]) and incubated with an anti-Alexa Fluor 488 monoclonal antibody (10 μg/ml) for 60 min at 37°C. Finally, PC12 cells were washed, fixed and mounted in Mowiol.

### Anti-flag labeling

The mouse monoclonal anti-Flag antibody (2.2 mg/ml) was incubated in 0.1 M bicarbonate buffer, pH 9.0, in the presence of Alexa Fluor 488 5*-*TFP (1 mg/ml) for 60 min at room temperature with gentle stirring with a vortexer. Next, the labeled antibody was purified by gel filtration (Bio-Gel P30, Bio-Rad) in Bio-Spin columns (Bio-Rad).

To measure the fluorescence associated with the perinuclear region of PC12 cells, a concentric circle was drawn using the smallest diameter of the cell with the ImageJ program (National Institutes of Health, USA). Inside this circle, another concentric circle, with half the diameter of the larger circle, was drawn. The fluorescence associated with the smallest circle was considered the perinuclear-associated fluorescence.

To calculate the endosomal volume of TrkA-positive endosomes, a tridimensional reconstruction using the ImageJ program was performed for each cell from Z-Stacks obtained with a confocal microscope. The endosomal volume was calculated by multiplying the larger diameter, the smaller diameter and the thickness of each endosome.

For transferrin loading of PC12 cells, cells were serum-starved for 90 min in incubation media and treated for 2 hrs at 37°C with Alexa Fluor 568-labeled transferrin (10 μg/ml or 60 μg/ml) in the same culture medium. Cells were then rinsed with PBS, fixed and mounted in Mowiol.

### Kinetics of NGF-activated signaling pathways in control and PC12-U18666A cells

PC12 cells at high density (70%) were grown on 3-cm plates in complete media and treated with U18666A (2 μg/ml) for 24 hrs. PC12 cells were then serum-starved for 2 hrs in DMEM/glutamine/high glucose-containing BSA (1 mg/ml) and treated for different durations (0-360 min) in the same media with NGF (5 ng/ml) in the presence of U18666A. To stop the treatment, PC12 cells were rinsed with cold PBS and lysed for 30 min in ice-cold lysis buffer [HEPES (50 mM), NaCl (150 mM), EGTA (1 mM), MgCl_2 _(5 mM), glycerol (10%) and Triton X-100 (1%)] supplemented with protease and phosphatase inhibitor cocktails. The PC12 supernatant was obtained after centrifugation, and proteins were measured using the Bio-Rad DC protein assay. Western blotting was performed with antibodies specific for phospho- and total Akt, ERK1/2 and PLC. Detection was performed with HRP-conjugated secondary antibodies and the West Pico ECL kit (Pierce). To measure ChAT, pAkt and Akt levels in the brain, the medial septum of WT and *NPC1*^-/- ^mice was micro-dissected and homogenized in cold lysis buffer supplemented with protease and phosphatase inhibitor cocktails. The brain tissue supernatant was obtained after centrifugation, and proteins were measured with the Bio-Rad DC protein assay. Western blotting was performed with specific antibodies, and detection was carried out with HRP-conjugated secondary antibodies and the West Pico ECL kit (Pierce).

### TrkA immunoprecipitation

PC12 cells seeded in 10-cm plates were treated as described above for the activation of NGF signaling pathways. PC12 cells were then washed with ice-cold PBS and lysed with buffer containing Tris (20 mM) pH 8.0, NaCl (150 mM), Igepal (1%), glycerol (10%) and EDTA (2 mM) and supplemented with protease and phosphatase inhibitor cocktails. Immunoprecipitation was performed as described previously [39].

### Microscopy

Optical and fluorescent microscopy were performed with brain sections and PC12 cells. Most of the digitized images were obtained with an Olympus BX51 (Tokyo, Japan) optical microscope (20× objective) equipped with a CoolSnap-Pro digital camera (Media Cybernetics, Maryland, USA) and connected to an image analysis system based on the Image-Pro Express software, version 5.1.0.12 (Media Cybernetics). The pictures were analyzed with SigmaScan software (SPSS; Chicago, Illinois, USA). Panoramic views of the MS were obtained with a 20× objective. Amplified images were taken with a 63× objective. Image analysis of PC12-related pictures was performed with the public-domain Image J 1.43 software.

ChAT levels in septal cholinergic neurons were quantified in images digitized by an Olympus IX71 inverted microscope equipped with a QImaging QICAM Fast 1394 digital camera and connected to an image analysis system based on the Image-Pro Express software (version 6.3.0.531, Media Cybernetics). Images of the septal areas from three animals per treatment (two coronal sections per animal) were analyzed. Images were digitally inverted, and the integrated intensity was measured for each ChAT immunopositive cell using the ImageJ software. For each treatment, the values of integrated intensity were averaged and compared with Student's *t*-test over a total of 180 cells per treatment in the case of ChAT and 54-61 cells in the case of TrkA. The total intensities of the ChAT and TrkA immunohistochemical or immunofluorescent levels per cell were standardized by the total size of the neuron that was quantified.

Confocal images from p75-labeled brain sections were collected using a Zeiss LSM Pascal 5 [including a triple laser module (Arg 458/488/514 nm, HeNe 543 nm, HeNe 633 nm; Carl Zeiss, Thornwood, NY)] connected to an inverted microscope (Axiovert 2000) with a 63× objective. Confocal images of ChAT-, NeuroTrace- and Fluoro-Jade C-labeled brain sections from axotomized brains were collected with a Olympus FluoView 1000 with a filter-type detector confocal microscope mounted on an inverted IX81 motorized microscope. Pictures of the septal areas from three animals per treatment (two coronal sections per animal) were analyzed to measure the intensity of ChAT staining and to quantify the number of NeuroTrace-positive neurons. For ChAT staining, images were digitally inverted, and the integrated intensity was measured for each ChAT-immunopositive cell with the ImageJ software. For each treatment, the values of the integrated intensities were averaged and compared with Student's *t*-test over a total of 92 ChAT-positive cells in the contralateral side of the axotomy and 40-43 ChAT-positive cells in the ipsilateral side of the axotomy. The number of neurons positive for NeuroTrace was manually counted from the digitized images. Only the cells that were inside a grid as shown in Additional file 1: Figure S1 were considered.

### Data analysis of brain sections and PC12-related experiments

Comparisons between the axotomized and unlesioned sides of the septum were statistically validated by Student's *t*-test to determine the level of significance (*p *< 0.05). The analyses were performed using the septum contralateral to the lesioned side as a control (100%).

Comparisons between control and PC12-U18666A cells were statistically validated by Student's *t*-test (or two-way ANOVA) to determine the level of significance, as indicated in the figure legends.

## Results

We used two different models to assess whether the NPC1 deficiency and endosomal abnormalities caused by cholesterol accumulation in the late endocytic pathway disrupted NGF signaling. First, we studied NGF signaling in vivo using the septo-hippocampal pathway, which responds to NGF after axotomy, by stabilizing the levels of choline acetyl transferase (ChAT) in axotomized cholinergic neurons. In addition, we established a cell culture model of NPC by treating PC12 cells with the drug U18666A.

**Figure 1:**
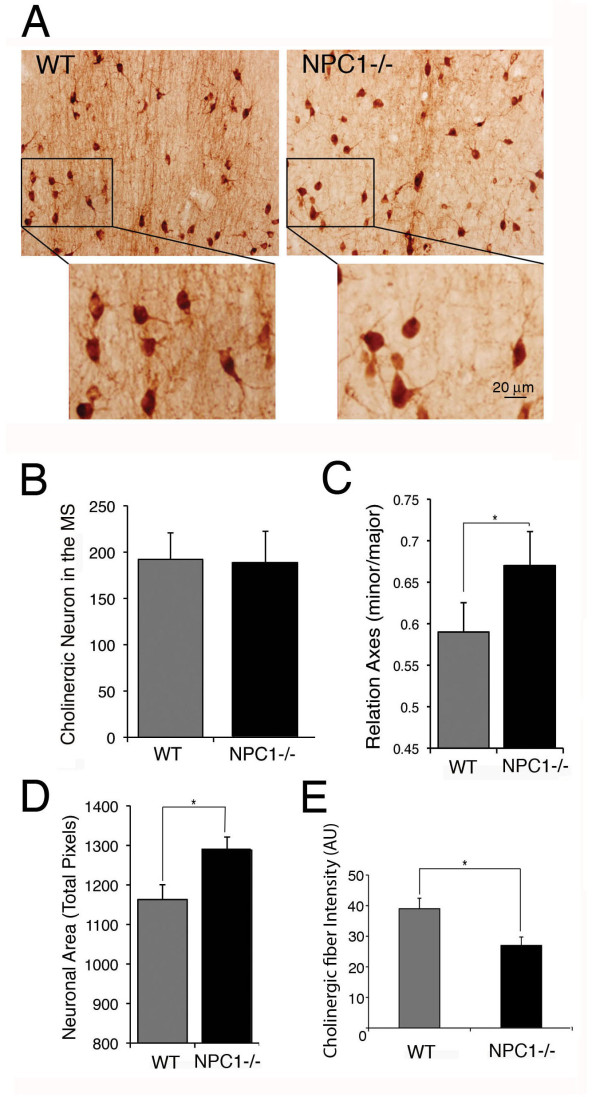
**Characterization of *NPC1*^-/- ^septal cholinergic neurons**. **A**. Brain sections at the level of the medial septum (MS) from 8-week-old WT and *NPC1*^-/- ^mice were stained for ChAT and visualized with secondary antibodies conjugated to HRP. The insets show a magnification of the cholinergic cells in the upper panels. WT cholinergic neurons are smaller and less rounded than *NPC1*^-/- ^cholinergic neurons, and there is a reduction in the number of cholinergic fibers surrounding the labeled *NPC1*^-/- ^cholinergic cells. **B**. The number of ChAT-labeled cholinergic neurons was quantified in the septal area of WT and *NPC1*^-/- ^brain sections. Four sections from six WT and six *NPC1*^-/- ^age-matched mice were used for quantification. There are no differences in the number of septal cholinergic neurons, p = 0.829. **C and D**. The morphology of septal cholinergic neurons was evaluated in ChAT-stained brain sections from WT and *NPC1*^-/- ^mice. *NPC1*^-/- ^cells have an increased area and exhibit a higher ratio between the minor and major axes of cholinergic neurons, * *p *< 0.006. A total of 600 cells from six WT and six *NPC1*^-/- ^age-matched mice were used for quantification. **E**. Quantification of p75-labeled cholinergic fibers in different septal regions without cholinergic neuronal somas, as shown in the insets of Additional file 2: Figure 2A, *p < 0.0001.

We used axotomy of the septo-hippocampal pathway with infusion of NGF by osmotic pumps to assess whether NGF signaling was altered in *NPC1-/- *mice. Loss of the cholinergic phenotype in cholinergic neurons after axotomy can be prevented by the intracerebroventricular infusion of NGF [40]. NGF receptors depend on endosomal transport for proper signaling, and endosomal function is heavily altered by NPC [6,41]. Cholinergic neurons from the MS of WT and *NPC1-/- *mice were visualized by immunohistochemistry against choline-acetyl transferase (ChAT) and p75. Although the number of cholinergic neurons in the MS remained unchanged (Figures 1A and 1B), the morphology of MS cholinergic neurons was clearly distorted (Figure 1A). Neurons appeared to be rounder and hypertrophic (Figure 1C and 1D), with reduced densities of proximal and distal fibers (Figure 1E). Cholinergic fibers in the MS were labeled with an antibody against p75 because this antibody better defines the cholinergic fibers compared to ChAT staining (Figure 1A and Additional file 2: Figure S2A). In addition to cholinergic fiber abnormalities, we also observed a five-fold increase in axonal spheroid staining for neurofilaments in the MS (Additional file 2: Figure S2B). Axonal spheroids are focal axonal swellings filled with organelles and cytoskeletal proteins and are one of the first neuropathological hallmarks of axonal disease [42]. These morphological abnormalities correlate with the fact that *NPC1-/- *cholinergic cells of the MS were loaded with cholesterol (Additional file 3: Figure S3), confirming that MS cholinergic neurons are affected by NPC1 deficiency.

**Figure 2:**
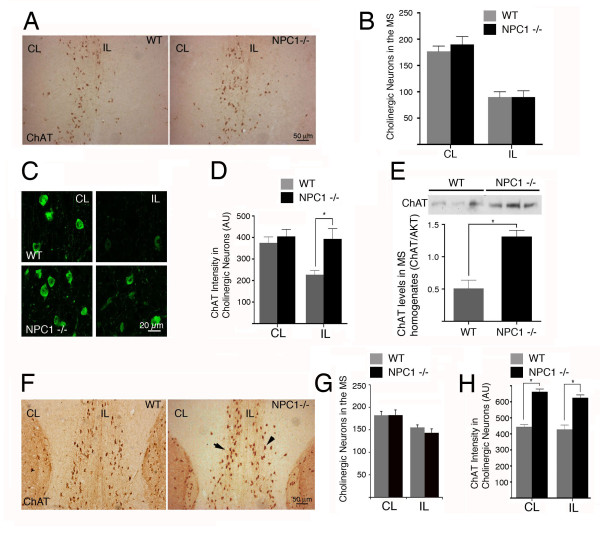
**In vivo NGF response in WT and NPC1-/- mice**. A and B. WT and *NPC1*^-/- ^mice respond similarly to axotomy of the fimbria-fornix. Brain sections from 8-week-old axotomized (unilateral fimbria-fornix axotomy) WT and *NPC1*^-/- ^mice were stained with an antibody against ChAT (A). Quantification shows an evident and similar reduction in the number of neurons labeled with ChAT (B). The differences between the phenotypes are not significant, *p *= 0.755. **C**. Brain sections from 8-week-old axotomized WT and *NPC1*^-/- ^mice were immunostained for ChAT, and the images were acquired by confocal microscopy. In WT mice, axotomized septal neurons (IL, ipsilateral to the axotomy) exhibit lower levels of ChAT than contralateral neurons (CL). In *NPC1*^-/- ^mice, ChAT staining in the axotomized septal neurons is similar to that in the contralateral neurons. **D**. The quantification of ChAT intensity in septal neurons (90-92 neurons for contralateral side and 40-42 neurons for ipsilateral) from three WT and three *NPC1*^-/- ^mice demonstrates a significant reduction in the ChAT levels in WT axotomized neurons, while *NPC1*^-/- ^axotomized neurons appear protected, *p < 0.005, unpaired one-way ANOVA. The increased staining of ChAT-labeled cells in C and D is not due to increased cell size because the total fluorescence or immunostaining intensity of each cell was normalized by cell area. **E**. Upper panel: ChAT levels were analyzed by western blot using homogenates of the MS from three 8-week-old WT and three *NPC1*^-/- ^mice. Bands corresponding to ChAT from three different WT and *NPC1*^-/- ^mice are shown. Lower panel: quantification of the western blot indicates that ChAT levels are significantly higher in *NPC1*^-/- ^mice than in WT mice. *p < 0.01, unpaired Student's *t*-test. n = 3 animals per phenotype. ChAT was normalized against total Akt. **F-H. Increased ChAT staining in response to NGF infusion in *NPC1*^-/- ^septal cholinergic neurons**. Brain sections from 8-week-old axotomized WT and *NPC1*^-/- ^mice infused with NGF for one week (F). Black arrows indicate cholinergic neurons with increased ChAT staining. Quantification shows that NGF protected septal cholinergic neurons equally in WT and *NPC1*^-/- ^mice (G). Quantification of ChAT staining in cholinergic cells (as indicated by the arrows in F). AU, arbitrary units. The differences are statistically significant, *p < 0.0001. Two sections through the medial septum of four WT and four *NPC1*^-/- ^age-matched mice were used for quantification. A total of approximately 180 cells were considered for each phenotype (H).

As shown previously in rats and mice [43-45], axotomy of the septal cholinergic neurons in WT and *NPC1*^-/- ^mice caused an approximately 50% loss of ChAT-positive neurons (Figure 2A and 2B) and an approximately 70% reduction of total cholinergic innervation, as shown by acetylcholinesterase (AChE) staining in the hippocampal side ipsilateral to the brain lesion (Additional file 4: Figure S4C). In addition, we analyzed the AChE reduction in specific regions of the hippocampus and observed that there was a greater reduction of cholinergic axons in *NPC1*^-/- ^mice compared to WT mice in the DG and the CA1b region of the hippocampus (Additional file 4: Figure S4E). We also quantified the total number of neurons in the MS with a general neuron-specific stain to investigate whether any other population of neurons in the MS was affected by the axotomy. The total level of neurons was similar in WT and *NPC1*^-/- ^mice, and no staining was evident with Fluoro-Jade, a histochemical stain for degenerating neurons [46] (Additional file 1: Figure S1).

These observations indicate that although *NPC1*^-/- ^cholinergic neurons had morphological abnormalities, they were not more vulnerable to axotomy than WT neurons. These results are consistent with our recently published observations showing a lack of degeneration of MS cholinergic neurons after axotomy in rats [40]. Although axotomy causes similar cholinergic neuronal loss in the MS of WT and NPC1-/- mice, we quantified the intensity of ChAT immunofluorescence from cholinergic cells in the contralateral and ipsilateral sides of the MS 7 days after axotomy. We observed that the cholinergic cells of the contralateral side of the axotomy had similar levels of ChAT staining in *NPC1*^-/- ^mice and WT mice; however, the cholinergic cells of the ipsilateral side of the lesion in the MS had increased staining for ChAT in *NPC1*^-/- ^mice compared to WT mice (Figure 2C and 2D). We also observed increased ChAT levels in MS homogenates from *NPC1-/- *mice compared to WT mice (Figure 2E). Because *chat *is under the control of NGF signaling, these results suggest an increased response to endogenous NGF [47,48]. Finally, to determine whether axotomized MS cholinergic neurons in *NPC1*^-/- ^mice respond to NGF in vivo, we infused WT and *NPC1*^-/- ^mouse brains with NGF for 7 days starting after the surgery. Surprisingly, *NPC1*^-/- ^cholinergic neurons responded similarly to WT neurons in response to the lesion and NGF treatment, with protection of approximately 80% of axotomized cholinergic neurons (Figures 2F and 2G). The reduction of AChE staining in the whole hippocampus was similar in WT and *NPC1*^-/- ^mice (Additional file 4: Figure S4D and F). However, consistent with the suggestion that *NPC-/- *cholinergic neurons have an increased response to NGF, the axotomized cholinergic neurons from NGF-infused *NPC1*^-/- ^mice exhibited increased ChAT staining compared to WT in the contralateral and ipsilateral sides of the lesions (Figure 2F and 2H). Because we found similar levels of AChE in the hippocampus of non-axotomized *NPC1*^-/- ^mice (Additional file 4: Figure S4A and B), we hypothesize that the increase in ChAT after NGF infusion was not due to altered anterograde axonal transport in cholinergic cells from *NPC1*^-/- ^mice but to an increased response to exogenous NGF. Altogether, these results suggest that cholinergic neurons from *NPC1*^-/- ^mice are more sensitized to NGF than those from WT mice.

To further elucidate the mechanism by which *NPC1*^-/- ^cholinergic neurons are sensitized to NGF signaling, we established a cell culture model of NPC by treating PC12 cells with the drug U18666A. PC12 cells are a good model in which to study NGF signaling because they express p75 and TrkA and respond to NGF by differentiating into a sympathetic-like neuron [30,31]. Treating PC12 cells with increasing concentrations of U18666A for 24 hrs resulted in a gradual accumulation of free cholesterol inside the cells, as measured by filipin staining. In addition, similarly to other NPC1 cellular models, PC12 cells treated with U18666A (PC12-U18666A) exhibited cholesterol and ganglioside GM1 accumulation and increased cell size as a result of cholesterol overload. Similarly to other NPC1 models, PC12-U18666A exhibited a change in the distribution pattern of late endosomes, yielding a structure similar to a ring filled with free cholesterol and with reduced mobility (Additional file 5: Figure S5, Additional file 6: Figure S6) [13,16,49-52]. All of these results indicate that PC12 cells treated with the drug U18666A are a good model for assessing the different cellular and molecular consequences of the NPC1 phenotype related to NGF signaling.

**Figure 3:**
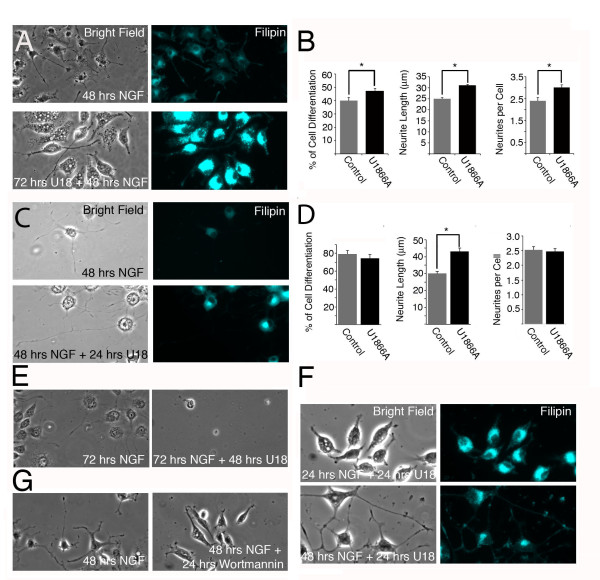
**Increased neurite length in NGF-treated PC12-U18666A compared to controls**. **A**. PC12 cells were treated for 24 hrs with 2 μg/ml of U18666A and for another 48 hrs with the same dose of the drug in the presence of NGF (5 ng/ml). After the treatments, PC12 cells were fixed and stained for filipin. **B**. The number of differentiated cells (cells with a neurite at least twice the diameter of the cell body), the length of the neurites and the number of neurites per cell were quantified in control and PC12-U18666A cells. A total of 100 cells from three different experiments were quantified. The differences between treatments are significant, *p < 0.02. **C**. PC12 cells were treated for 24 hrs with NGF (5 ng/ml) and for an additional 24 hrs with NGF alone (48 hrs NGF) or in the presence of U18666A (2 μg/ml) (48 hrs NGF + 24 hrs U18). **D**. The number of differentiated cells, the length of neurites and the number of neurites per cell were quantified in the conditions defined in C. The differences between treatments are significant, *p < 0.02. **E**. PC12 cells were treated for 72 hrs with NGF (72 hrs NGF) or 24 hrs with NGF followed by 48 hrs with U18666A (72 hrs NGF + 48 hrs U18). Cells treated with the drug are stained, as shown in the representative images. **F**. PC12 cells were treated for 48 hrs with NGF (48 hrs NGF) or 24 hrs with NGF followed by 24 hrs with U18666A in the absence of NGF (24 hrs NGF + 24 hrs U18). PC12 cells treated with the drug in the absence of NGF retracted neuritis, which is evident compared with cells that were treated with NGF for 48 hrs. **G**. PC12 cells were treated for 48 hrs with NGF (48 hrs NGF) or for 24 hrs with NGF followed by 24 hrs with wortmannin (100 nM) in the presence of NGF. PC12 cells treated with wortmannin retracted neuritis, indicating that neurite elongation is at least partly regulated by IP3K signaling.

To assess whether the NPC1 phenotype is translated into morphological alterations in PC12-U18666A cells, we differentiated PC12-U18666A cells with NGF for two days. We observed an increased number of differentiated cells in PC12-U18666A cells compared to controls (Figures 3A and 3B). In addition, PC12-U18666A cells had more and longer neurites per cell (Figure 3B). To assess the effect of U18666A in neurite outgrowth and to preclude the effect of differentiation, we first differentiated naïve PC12 cells with NGF and then treated the cells with NGF alone or in the presence of the drug for another 24 hrs. Under these conditions, we did not observe a difference in the number of differentiated cells (cells with a neurite at least twice as long as the diameter of the cell body) or neurites, but neurite length increased significantly (Figure 3C and 3D). PC12 cells that were first treated with NGF for 24 hrs and then treated for an additional 48 hrs with U18666A in the presence of NGF did not survive (Figure 3E), in contrast to PC12-U18666A treated for 48 hrs with NGF, indicating that the increased neurite effect observed in Figures 3C and 3D did not lead to survival but was part of a degenerative process. To eliminate the possibility that the effect of U18666A on neurite length was due to the drug itself and not the NGF response, we differentiated PC12 cells with NGF for 24 hrs and then treated the cells with the drug alone or with NGF for another 24 hrs. We observed that PC12 cells treated with the drug alone retracted their neurites, a change that was evident when compared to the PC12 cells that were treated with NGF (Figure 3F). Finally, to assess whether the neurite growth process was due to IP3K-Akt signaling (see below), we treated NGF-treated PC12 cells for 24 hrs with NGF or with NGF plus wortmannin (a specific inhibitor of IP3K). We observed that PC12 cells treated for 24 hrs with wortmannin (in the presence of NGF) retracted their neurites compared to PC12 cells that were treated only with NGF (Figure 3F). These results suggested that PC12-U18666A, similar to *NPC1-/- *cholinergic neurons, are sensitized to NGF, leading to increased neurite length, a process that is related to degeneration and not survival.

**Figure 4:**
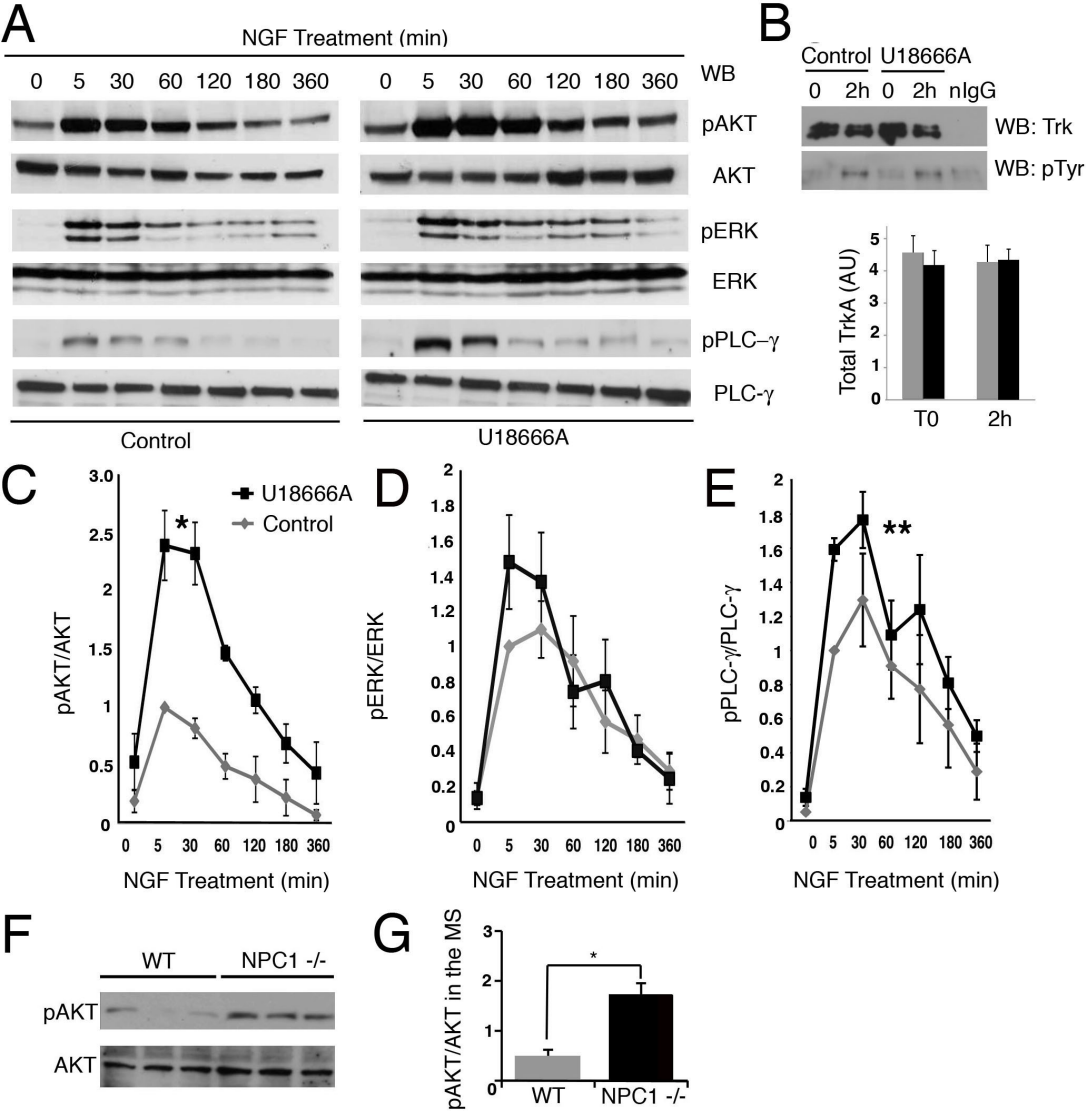
**Increased Akt activation in NGF-treated PC12-U18666A and in the medial septum of *NPC1*^-/- ^mice compared to WT**. **A**. PC12 cells were treated for 24 hrs with 2 μg/ml U18666A and with 5 ng/mL NGF for different durations (0-360 min). After NGF treatment, PC12 cells were homogenized, and the samples were processed for SDS-PAGE, blotted and immunolabeled for phosphorylated Akt (pAKT), total Akt (AKT), phosphorylated ERK1/2 (pERK), total ERK1/2 (ERK), phosphorylated PLC-γ (pPLC-γ) and total PLC-γ (PLC-γ). **B**. PC12 cells were treated for 24 hrs with 2 μg/ml U18666A (U18666A) or not treated (Control) and then were left untreated (T0) or incubated with NGF for 2 hrs (2 h). After treatment, PC12 cells were homogenized, followed by immunoprecipitation with a polyclonal antibody against Trk (C14, Santa Cruz) and immunoblotting for total Trk (upper panel, monoclonal B3, Santa Cruz) or with a monoclonal antibody against phosphotyrosine (pTyr). The graph shows the quantification of total TrkA in control and PC12-U18666A cells at time 0 (T0) or after 2 hrs of NGF treatment (2 h). The levels of TrkA are equivalent in both experimental groups. **C**. The graph shows the quantification of pAkt normalized to total Akt ± SEM from three different experiments. The differences between treatments are significant, *p < 0.001 (two-way ANOVA). **D**. The graph shows the quantification of activated ERK1/2 (pERK) normalized to total ERK ± SEM from five different experiments. The differences between treatments are not significant (two-way ANOVA). **E**. The graph shows the quantification of activated PLC-γ (pPLC-γ) normalized against total PLC-γ ± SEM from four different experiments. The differences between treatments are significant, **p < 0.05, two-way ANOVA. **F**. Levels of phosphorylated Akt (pAKT) and total Akt (AKT) were analyzed by western blot of homogenates of the MS of three 8-week-old WT and three 8-week-old *NPC1*^-/- ^mice. Bands corresponding to pAkt and Akt from three different WT and *NPC1*^-/- ^mice are shown. **G**. Quantification of the western blot shown in F indicates that pAkt levels are significantly higher in *NPC1*^-/- ^mice than in WT mice, *p < 0.01, unpaired Student's *t*-test. n = 3 animals per phenotype. pAkt levels were normalized against total Akt.

To assess whether the endosomal abnormalities translate into misregulation of receptor signaling, which could explain increased neurite length, we measured the activation of different signaling pathways downstream of TrkA, such as ERK1/2, PI3K/Akt and PLC-γ. Although there were similar basal levels of Akt and PLC-γ in PC12-U18666A and control cells, after 5 min of NGF treatment, there was an approximately 150% increase in Akt phosphorylation and a 40% increase in active PLC-γ in PC12-U18666A cells compared to the controls (Figures 4A, C and 4E). The increase in Akt phosphorylation lasted for up to 2 hrs after NGF addition, returning to basal levels just after 3 hrs (Figures 4A and 4C). This increased response to NGF was not due to increased levels of total TrkA or to a slower down-regulation of the receptor because the total TrkA level was the same immediately following NGF addition and 2 hrs later (Figure 4B). In addition, a similar level of TrkA phosphorylation was observed after 2 hrs of NGF treatment (Figure 4B). These results indicate that PC12-U18666A cells had increased Akt phosphorylation after NGF addition, as characterized by the up-regulation of Akt activity over a longer time, than that observed in the control cells. NGF also significantly increased PLC-γ activation in PC12-U18666A cells compared to the controls. However, these results were less robust than the results obtained for Akt. Consistently, when the levels of pAkt were measured in homogenates of the medial septum (MS) (where NGF-responsive neurons are located), we observed increased pAkt levels in the MS of *NPC1 -/- *mice compared to WT mice (Figure 4F and 4G). This result suggests that NGF-responsive neurons may be more sensitive to NGF, particularly Akt signaling, compared with WT mice or normal conditions (naïve PC12 cells). Unlike Akt and PLC-γ, ERK1/2 activation was not significantly up-regulated in PC12-U18666A cells compared to control cells (Figures 4A and 4D). These results suggest that the up-regulation of the Akt signaling pathway may be responsible for the increased neurite length exhibited by PC12 cells treated with U18666A.

**Figure 5:**
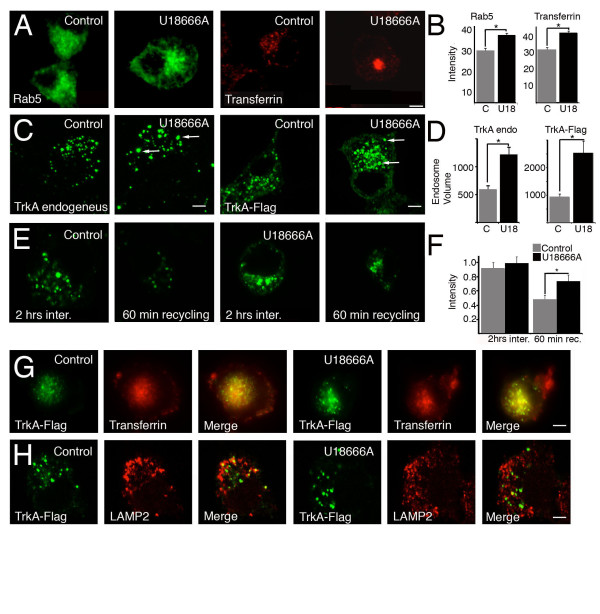
**Transferrin-positive endosome abnormalities and reduced recycling of the TrkA receptor in PC12-U18666A cells**. **A**. PC12 cells were transfected with a Rab5-EGFP plasmid (which labels early endosomes in green) and after 24 hrs were treated (U18666A) or not treated (Control) with U18666A (2 μg/ml) for another 24 hrs. To load the cells with transferrin (labeling recycling endosomes in red), PC12 cells were serum-starved for 1 hr and treated with transferrin Alexa Fluor 555 (2 μg/ml) for 2 hrs at 37°C. Cells were fixed with paraformaldehyde and visualized with a confocal microscope. Scale bar, 5 μm. **B**. The fluorescence from Rab5-EGFP (n = 16-24 cells, from two different experiments) and transferrin (n = 57-60 cells, from two different experiments) associated with the perinuclear region of PC12 cells was measured and plotted. C, control PC12 cells. U18, PC12 cells treated for 24 hrs with U18666A (2 μg/ml), *p < 0.0001. **C**. Immunoendocytosis (2 hrs at 37°C) of endogenous TrkA (TrkA endogenous) and TrkA-Flag in PC12 cells not treated (Control) or treated (U18666A) for two days with the drug (2 μg/ml). The arrows indicate the presence of larger endosomes in U18666A-treated PC12 cells than in non-treated cells. **D**. Volume quantification of TrkA-positive endosomes of PC12 cells non-treated (C) or treated (U18) with U18666A (2 μg/ml). For endogenous TrkA-labeled endosomes (TrkA endo), a total of 63-69 vesicles were considered (from 6-7 representative cells from two different experiments). *p < 0.0001. For TrkA-Flag-labeled endosomes, a total of 15-19 endosomes were considered (from 2 representative cells). *p < 0.0003. **E**. Immunoendocytosis of TrkA-Flag was allowed to proceed for 2 hrs at 37°C in the presence of NGF (2 hrs inter). The Alexa Fluor 488-conjugated anti-Flag monoclonal antibody was then washed from PC12 cells by treatment with cooled EDTA. PC12 cells were fixed and mounted. For recycling experiments, immunoendocytosis of TrkA-Flag was allowed to proceed for 60 min, and the Alexa Fluor 488-conjugated anti-Flag monoclonal antibody was then washed from PC12 cells by treatment with cooled EDTA and further incubated with an anti-Alexa Fluor 488 antibody for 60 min at 37°C (60 min recycling), fixed and mounted. **F**. The fluorescence associated with PC12 cells after the treatments indicated in E was quantified and plotted. 2 hrs inter, 2 hrs internalization. 60 min rec, 60 min recycling. To measure the fluorescence associated with PC12 cells after 2 hrs of internalization of the Alexa Fluor 488-conjugated anti-Flag monoclonal antibody, 61-67 cells were considered from two different experiments. To measure the fluorescence associated with PC12 cells after 60 min recycling, 23-45 cells were considered from two different experiments. *p < 0.0023. **G**. To visualize the co-localization of TrkA-Flag with transferrin, PC12 cells not treated (control) or treated (U18666A) with the drug were incubated with the Alexa Fluor 488-conjugated anti-Flag monoclonal antibody and Alexa Fluor 555-conjugated transferrin (60 μg/ml) for 2 hrs in the presence of NGF and fixed. **H**. To visualize the co-localization of TrkA-Flag with LAMP2, PC12 cells not treated (control) or treated (U18666A) with U18666A were incubated with the Alexa Fluor 488-conjugated anti-Flag monoclonal antibody for 2 hrs in the presence of NGF, fixed and immunostained with a polyclonal antibody against LAMP2 (lysosomal marker).

Finally, we investigated in greater detail the endosomal abnormalities produced by U18666A treatment of PC12 cells and the trafficking properties of the TrkA receptor. In addition to changes observed in the distribution of late endosomes (Additional file 5: Figure S5 and Additional file 6: Figure S6), we also observed changes in the distributions of other endosomal populations. In PC12-U18666A cells, the Rab5-EGFP-positive endosomes (early endosomes) and transferrin-positive endosomes (recycling endosomes) exhibited perinuclear localization rather than peripheral localization, as in untreated cells (Figure 5A and 5B). This change in endosomal distribution could reflect changes in the Rab-GTPases and motor activity, as previously suggested by Gruenberg et al. [15]. To further assess whether these endosomal abnormalities are translated into changes in the trafficking capabilities of the TrkA receptor, we studied the internalization and recycling of the TrkA receptor as previously reported [38,39]. We found that TrkA-positive endosomes were larger in size in PC12-U18666A cells than in control cells (Figure 5C and 5D), and although PC12-U18666A cells and control cells showed similar levels of internalization, recycling of the TrkA receptor was reduced in PC12-U18666A compared to control cells (Figure 5E and 5F). The reduced recycling of TrkA was not due to missorting of the receptor to another intracellular route because after 2 hrs of internalization in the presence of NGF, most TrkA receptors were located in the recycling pathway and not the degradative pathway in both PC12-U18666A cells and control cells (Figure 5G and 5H). These results suggest that the abnormalities in endosomal dynamics observed in PC12-U18666A cells and other cellular models may contribute to the misregulation of receptor signaling in the endocytic pathway. To assess whether PC12-U18666A differentiated with NGF showed any sign of a degenerative process, we stained the cells with the AT8 antibody, which labels hyperphosphorylated tau protein. Increased phosphorylation of tau, as measured with the AT8 antibody, has been demonstrated previously by western blot in the brain of *NPC1*^-/- ^mice [53]. Differentiated PC12-U18666A cells showed increased AT8 immunostaining that was distributed throughout the cell, including in neurites (Additional file 7: Figure S7).

Taken together, our results suggest that *NPC1*^-/- ^cholinergic cells are sensitized to NGF compared to WT neurons, which correlates with the increased NGF response in PC12-U18666A. In addition, these results show that *NPC1*^-/-^cholinergic neurons, despite obvious morphological abnormalities, were able to respond to NGF in vivo.

## Discussion

Our study emphasizes the role of anomalous neurotrophin signaling in the neuropathology of NPC and is the first to demonstrate neuropathological changes in the septal cholinergic system of the basal forebrain in *NPC1*^-/- ^mice. Although several kinases are up-regulated in NPC brains [54-57], to the best of our knowledge, this is the first time that up-regulation of a kinase, particularly Akt, has been linked to a specific signaling transduction pathway such as NGF signaling in NPC.

The goal of our research was to investigate whether NGF signaling is affected in NPC1 disease in two models known to respond to NGF. Initially, we analyzed NGF signaling in vivo by infusing the brains of axotomized *NPC1*^-/- ^and WT mice with NGF. MS cholinergic neurons from *NPC1*^-/- ^mice responded to NGF in vivo and had increased ChAT staining after NGF infusion compared to WT mice. Although the increased expression of ChAT was readily evident in NGF-infused mice, increased ChAT expression was also detected in the presence of endogenous levels of NGF in the ipsilateral side of axotomized *NPC1*^-/- ^mice compared to the WT mice. In this case, ChAT levels were measured by immunofluorescence; however, increased ChAT levels were also evident in non-axotomized *NPC1*^-/- ^mice when the ChAT levels in MS homogenates from *NPC1*^-/- ^mice were compared to WT mice by western blotting. We also found an increased amount of phosphorylated Akt in the medial septum of non-axotomized *NPC1*^-/- ^mice compared to WT mice. These observations support the regulation of ChAT gene transcription by TrkA activation, particularly through the Akt signaling pathway, as described previously [47,48]. The increased Akt phosphorylation found in NGF-treated PC12-U18666A cells (see below) may partly explain the increased ChAT levels in *NPC1*^-/- ^MS cholinergic neurons.

The conserved response to NGF in *NPC1*^-/- ^mice was surprising because we found that the morphological alterations of MS cholinergic neurons in *NPC1*^-/- ^mice were similar to those of neurons from different brain regions, such as brain stem neurons, of patients with NPC disease [58]. Purkinje cell loss is a prominent feature of the *NPC1*^-/- ^mouse model, and some Purkinje cells exhibit a severely stunted or retracted dendritic arbor before neuronal death [59]. These observations, together with our findings in MS cholinergic neurons, suggest that dendritic alterations precede neuronal loss as a general feature of neurodegeneration in the NPC brain. These results also explain the reduced vulnerability of cholinergic neurons compared to Purkinje cells because cholinergic neurons receive additional trophic support from hippocampal neurons and oligodendrocytes [60-63]. Although cholinergic neurons may be more protected than Purkinje cells temporarily, the chronic up-regulation of NGF signaling pathways, such as Akt, may lead to abnormal phosphorylation of cytoskeletal proteins. Consistent with this idea, several kinases, including ERK1/2, CDK5, c-Abl, and PI3K, are up-regulated in the brains of *NPC1*^-/- ^mice [54-57].

To gain greater insight into the effect of NPC1 inhibition and cholesterol overload in the endocytic pathway on NGF signaling, we established a cell culture model of NPC by treating PC12 cells with the drug U18666A. As expected, PC12-U18666A cells fully recapitulated the key characteristics of the cellular phenotype of cells from NPC patients and cellular models of NPC disease, such as increased cholesterol, endosomal abnormalities and GM1 accumulation [50,64]. Although U18666A is a pharmacological tool and its use may induce other cellular changes that are not related to the down-regulation of NPC1 function (see review by [31]), we found that, at the cellular level, PC12-U18666A cells mirrored the phenotype of other NPC cellular models [64]. One general observation in PC12-U18666A cells was that there were profound changes in the distribution of endosomes. These changes could have been the result of the misregulation of Rab activities. Rab proteins are small monomeric GTPases that regulate many steps of membrane trafficking, including vesicle formation, vesicle movement along actin microfilament and microtubules and membrane fusion [65]. In cellular models of NPC disease, cholesterol overload influences the retrieval of Rab proteins (Rab4, Rab5, Rab9 and Rab7) from different endosomal membranes, which leads to the sequestration of Rabs in inactive forms [13-15] and disturbs the movement of vesicles along microtubules by influencing molecular motor activity [15]. Therefore, the changes in the endosomal distribution in PC12-U18666A cells may be related to altered transport and maturation of these endosomes as a result of the misregulation of motor proteins.

PC12-U18666A cells treated for two days with low concentrations of NGF exhibited greater differentiation and more and longer neurites than control cells, indicating that PC12-U18666A cells are more sensitized to NGF. This result correlated with an up-regulation of Akt and PLC-γ signaling but not ERK1/2 when PC12-U18666A cells were treated with NGF for short durations (15 min-6 hrs). Akt and PLC-γ signaling regulate neuronal morphology in different neuronal types, including PC12 cells, consistent with increased NGF-induced differentiation in PC12-U18666A cells [66-68]. The differentiation process in PC12 cells is measured by quantifying cells that possess neurites that are twice the diameter of the cell body. Therefore, it is difficult to separate the process of differentiation from the process of neurite growth. Therefore, we treated PC12 cells with U18666A then with NGF. Under these conditions, we observed increased neurite length but not increased differentiation. In addition, neurite growth of differentiated PC12 cells was inhibited by a specific inhibitor of the IP3K-Akt pathway (wortmannin). Taken together, these data suggest that the treatment of PC12 cells with U18666A increases IP3K-Akt signaling in response to NGF-induced increased neurite growth. Differentiated PC12 cells die after 48 hrs of U18666A treatment, and PC12-U18666A cells treated for 48 hrs with NGF show increased AT8 phosphorylation, suggesting that the increase in NGF signaling induced by U18666A treatment is related to a pathological process rather than a survival signal. The increase in Akt signaling in PC12-U18666A cells treated with NGF correlates with increased levels of phosphorylated Akt in the MS of *NPC1-/- *mice compared to WT mice. Cholinergic neurons located in the MS normally respond to NGF signaling by increasing ChAT synthesis, suggesting that the inhibition of NPC1 may also cause increased NGF-signaling in vivo.

The AT8 antibody is one of the most widely used antibodies against phosphorylated tau and recognizes neurofibrillary lesions in several neurodegenerative disorders [69]. Interestingly, up-regulation of Akt in AD brains has been correlated with the presence of AT8 immunoreactivity [70]. Taken together, these results suggest that, rather than inducing survival, the chronic up-regulation of signaling pathways such as Akt in post-mitotic neurons may ultimately misregulate other signaling pathways, leading to abnormal phosphorylation of the cytoskeleton and neuronal degeneration.

Several lines of evidence suggest that the abnormal recycling of receptors may play a role in abnormal signaling and trafficking of receptors in NPC. For example, the impairment of transferrin and lipid recycling has been observed in cellular NPC models [14], and the impairment of Rab function has been proposed to cause normal influx but slower efflux of components in the endocytic compartment [50,71]. Indeed, consistent with a receptor recycling problem in NPC cells, we found an abnormal distribution of early and recycling endosomes, increased TrkA-positive endosome size and reduced recycling of the TrkA receptor in PC12-U18666A cells. Along with the observation of Lee et al. [38] that NGF-induced long-lasting activation of Akt signaling depends on TrkA receptor recycling, our results suggest that the increased Akt activation in both NPC models may be due, at least in part, to reduced recycling of the TrkA receptor. This phenomenon could also explain why TrkB activation is inhibited in striatal neurons derived from *NPC1*^-/- ^mice; TrkB has reduced recycling capacities than TrkA and thus it is affected differently by the accumulation of cholesterol [12,38]. Or alternatively, TrkB may be more sensitive than TrkA to changes in the cholesterol content of the plasma membrane [72]. Both of these scenarios would give rise to different responses to downstream signaling by TrkA and TrkB in the context of the NPC phenotype [11].

## Conclusions

We have demonstrated for the first time that the endosomal alterations caused by NPC result in the up-regulation of a specific signaling pathway. Some of the neurodegenerative changes observed in NPC may be due to misregulation of the signaling networks that normally control proper neuronal physiological function.

## Competing interests

The authors declare that they have no competing interests.

## Authors' contributions

CC, AF, OML, CG, CP participated in the design and performed all of the experiments. FCB drafted the manuscript, conceived and coordinated the study and participated in the design of all of the experiments. NCI helped to coordinate the axotomy experiments and the manuscript drafting and critically reviewed the drafts. CGB, AK, AA, and SZ participated in the experimental design and critically reviewed the drafts. All authors read and approved the manuscript.

## Supplementary Material

Additional file 1 **Figure S1**. In the left upper panel, brain sections from 8-week-old axotomized WT and *NPC1*^-/- ^mice were double-stained with antibodies against ChAT (green) and the neuronal marker NeuroTrace (red). IL, ipsilateral side to the axotomy. CL, contralateral side to the axotomy. The ipsilateral side exhibits a reduction in the number of ChAT-positive neurons but no differences in the NeuroTrace staining. In the left lower panel, similar brain sections were probed for Fluoro-Jade C; no neurons were positive for this marker of neurodegeneration. Right panel, Brain sections from three WT and three *NPC1*^-/- ^mice were used to count total neurons in the medial septum; a scheme of the grid used for the quantification of total medial septum neurons is shown in the left panel. The quantification reveals a similar number of total neurons in both sides of WT and *NPC1*^-/- ^mice. Click here for file

Additional file 2 **Figure S2**. The MS of *NPC1*^-/- ^mice exhibits a decrease in p75-labeled cholinergic fibers and an increase in spheroids. **A**. Brain sections from 8-week-old WT and *NPC1*^-/- ^mice were stained for p75 and visualized with secondary antibodies conjugated to HRP. p75 was chosen because it labels cholinergic fibers better than the ChAT cholinergic marker. Both proteins are frequently used to label cholinergic cells in mice. The inset shows a magnification of the cholinergic fibers from the upper panels. In brain sections from *NPC1*^-/- ^mice, there are fewer fibers labeled with p75, and the fibers appear disrupted and less defined. The reduction of p75-labeled cholinergic fibers surrounding cholinergic cells is also obvious in this preparation. **B**. Brain sections from 8-week-old WT and *NPC1*^-/- ^mice were stained with an antibody against neurofilament. Arrows indicate axonal spheroids. The quantification indicates that there are more axonal spheroids in the medial septum of *NPC1*^-/- ^mice than in the medial septum of WT mice, *p < 0.0003. Three sections through the medial septum of four WT and four *NPC1*^-/- ^age-matched mice were used for the quantification of axonal spheroids. Click here for file

Additional file 3 **Figure S3**. Cholesterol overload in septal cholinergic neurons of *NPC1*^-/- ^mice. Brain sections from 8-week-old WT mice, shown in **A**, and *NPC1*^-/- ^mice, shown in **B**, were double-labeled with a polyclonal antibody against ChAT (labeling septal cholinergic cells, shown in green) and filipin (labeling cholesterol, shown in red). The arrows in B indicate cholinergic neurons that clearly demonstrate an accumulation of cholesterol in the soma. Click here for file

Additional file 4 **Figure S4**. Analysis of AChE staining in the hippocampus of *NPC1*^-/- ^WT mice. **A and B**. Hippocampal sections from 8-week-old WT and *NPC1*^-/- ^mice were stained for AChE (A). The comparison between WT and *NPC1*^-/- ^brains shows no major differences in the distribution of cholinergic terminals in the hippocampus (B). The different anatomic areas analyzed in B are indicated as CA1 (divided into layers a, b and c) and CA3 for the "cornu ammonis" and DG for the "dentate gyrus". The intensity of AChE was measured for each hippocampal region indicated in A and compared between six WT (gray bars) and six *NPC1*^-/- ^(black bars) mice. There were no significant differences in any of the studied regions (*p *≥ 0.19). The lower panel shows the intensity profile of a line traced along the dentate gyrus (also indicated in A). Differences in cholinergic terminal density are found only in the outer molecular layer of the DG (arrow), which is decreased in *NPC1*^-/- ^mice. **C and E**. Brain sections from axotomized *NPC1*^-/- ^mice and WT mice were stained for AChE (C). The quantification shows a differential reduction in AChE staining in cholinergic fibers according to the hippocampal region considered (as shown in A) in WT and *NPC1*^-/- ^mice one week after axotomy (E). For quantification, four sections of five different mice were considered, *p < 0.04. CL, contralateral side to the axotomy. IL, ipsilateral side to the axotomy. **D and F**. Brain sections from axotomized *NPC1*^-/- ^mice and WT mice were stained for AChE after one week of axotomy and concomitant NGF infusion (D). The quantification reveals similar AChE staining in the hippocampus, even after NGF treatment, in axotomized WT and *NPC1*^-/- ^mice, *p *= 0.2 (F). Four sections through the MS or the hippocampus of four WT and four *NPC1*^-/- ^age-matched mice were used for the quantification. CL, contralateral to the axotomy. IL, ipsilateral to the axotomy. ** The differences are not significant, *P *= 0.2. Click here for file

Additional file 5 **Figure S5**. PC12-U18666A cells have an NPC-like phenotype. Accumulation of cholesterol and GM1 ganglioside. **A**. PC12 cells were not treated (control) or treated with (2 μg/ml) U18666A for 24 hrs and stained with filipin (shown in red) before fixing with paraformaldehyde. **Left panels**, The fluorescence intensity profile of the red line drawn in the control and PC12-U18666A cells is shown. This result indicates that there is a difference in the distribution of cholesterol-labeled organelles. **B**. Quantification of total fluorescence intensity of filipin staining in control and U18666A-treated (2 μg/ml) PC12 cells. These results indicate that PC12-U18666A cells exhibit increased intracellular accumulation of cholesterol. **C**. Control or U1866A-treated PC12 cells were incubated with Alexa Fluor 555-conjugated cholera toxin subunit-B (CT-B) (20 μg/mL) for 30 minutes to label the accumulation of GM1 gangliosides in endosomes. The images were acquired under a confocal microscope. **Left panel**, There is a significant increase in the accumulation of GM1 in PC12-U18666A cells compared to control PC12 cells, *p < 0.0001, unpaired Student's *t*-test. n = 470-490 cells per treatment from two different experiments. **D**. PC12 cells were treated with 2 μg/ml U18666A for 24 hrs before staining with filipin (shown in red), fixing with paraformaldehyde and immunostaining for the lysosomal marker LAMP2 (shown in green). LAMP2 immunostaining indicates changes in the distribution of lysosomes from a perinuclear to a widespread distribution similar to the cholesterol-labeled organelles shown in A. **E**. The graph indicates an increase in the diameter of PC12-U18666A cells compared to control PC12 cells, *p *< 0.0001. Click here for file

Additional file 6 **Figure S6**. Rab7-positive endosomes are abnormal in PC12-U18666A cells. **A**. PC12 cells were transiently transfected with Rab7-EGFP (late endosome marker, shown in green) and, after 24 hrs, treated with 2 μg/ml U18666A (U18666A) for another 24 hrs before filipin staining (shown in red). The white arrow indicates a giant Rab7 endosome overloaded with cholesterol. It is also evident that the distribution of Rab7 vesicles (control) changed as a result of treatment with U18666A. **B**. PC12 cells were transfected with a Rab7-EGFP plasmid and treated with NGF for 24 hrs in the presence (U18666A) or absence (control) of U18666A, and the dynamics of Rab7-positive vesicles were compared. The movement of Rab7-positive vesicles was studied by confocal microscopy of living cells with a frequency of 100 frames/min. The first frames of the sequences for the control and PC12-U18666A cells are shown as insets in the upper panels. Then, we condensed 150 frames to 1 frame, shown in the upper panel. High levels of fluorescence in the projections are a consequence of the presence of static particles. In the lower panel, the same projections are shown but with segmentation of intensity ranges into a pseudo-colored scale. Sites with static endosomes are pink or yellow, and sites with more dynamic changes in intensity are green. Note that green areas are less apparent in PC12-U18666A cells, indicating that most of the Rab7-positive endosomes are static. **C**. Endosomes of five control and five PC12-U18666A cells were measured and categorized into three size ranges (smaller than 0.5 μm, between 0.5 and 1 μm and bigger than 1 μm). The plot shows a histogram of the endosome size distribution in control or PC12-U18666A cells. The relative abundance (frequency) of smaller endosomes is significantly decreased in U18666A-treated cells, while medium-sized endosomes are more abundant. PC12-U18666a cells also contain enlarged endosomes. The differences between treatments are significant, *p < 0.05. Click here for file

Additional file 7 **Figure S7**. Increased AT8 immunolabeling in differentiated PC12-U18666A cells compared to controls. **A**. PC12 cells were treated for 24 hrs with 2 μg/ml U18666A and for another 48 hrs with the same dose of the drug in the presence of NGF (5 ng/ml). After the treatments, PC12 cells were fixed and immunostained with the TAU5 monoclonal antibody, which labels total tau (green), or the AT8 monoclonal antibody against phosphorylated tau (red). PC12-U18666A cells have increased size and increased immunoreactivity against phosphorylated tau in the cell body and neurites. **B**. The intensity of the immunostaining of PC12 cells labeled with the TAU5 (which labels nonphosphorylated epitope in Tau) or AT8 antibody was quantified in the cell bodies of approximately 50 cells. The total fluorescence level of each cell was divided by the total area of the cell. The experiment was repeated three times with the same result, and one representative experiment was quantified. There was no significant difference in the fluorescence levels of TAU5 immunolabeling in control and PC12-U18666A cells, *p *= 0.6245. The differences in fluorescence levels of AT8-immunolabeling in control and PC12-U18666A cells are significant, *p < 0.005. Click here for file
