# Supplementary figures and images for: Cholinergic Abnormalities, Endosomal Alterations and Up-Regulation of Nerve Growth Factor Signaling in Niemann-Pick Type C Disease
Source: Mol Neurodegener. 2012 Mar 29;7:11. doi: 10.1186/1750-1326-7-11 (PMC3395862; doi:10.1186/1750-1326-7-11)

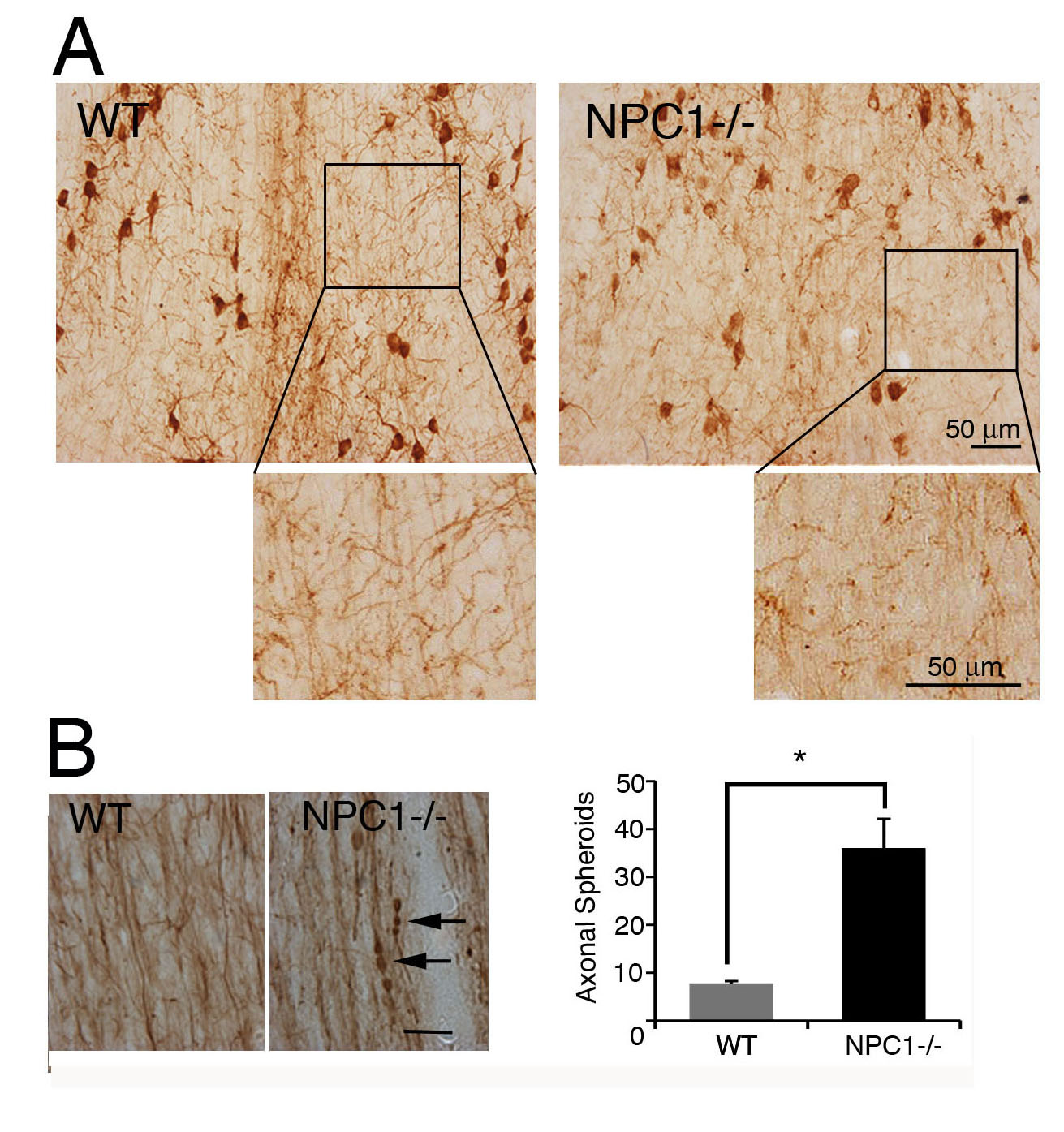

Supplement: Additional file 2 — Figure S2. The MS of NPC1-/- mice exhibits a decrease in p75-labeled cholinergic fibers and an increase in spheroids. A. Brain sections from 8-week-old WT and NPC1-/- mice were stained for p75 and visualized with secondary antibodies conjugated to HRP. p75 was chosen because it labels cholinergic fibers better than the ChAT cholinergic marker. Both proteins are frequently used to label cholinergic cells in mice. The inset shows a magnification of the cholinergic fibers from the upper panels. In brain sections from NPC1-/- mice, there are fewer fibers labeled with p75, and the fibers appear disrupted and less defined. The reduction of p75-labeled cholinergic fibers surrounding cholinergic cells is also obvious in this preparation. B. Brain sections from 8-week-old WT and NPC1-/- mice were stained with an antibody against neurofilament. Arrows indicate axonal spheroids. The quantification indicates that there are more axonal spheroids in the medial septum of NPC1-/- mice than in the medial septum of WT mice, *p < 0.0003. Three sections through the medial septum of four WT and four NPC1-/- age-matched mice were used for the quantification of axonal spheroids. [file 1750-1326-7-11-S2.JPEG]

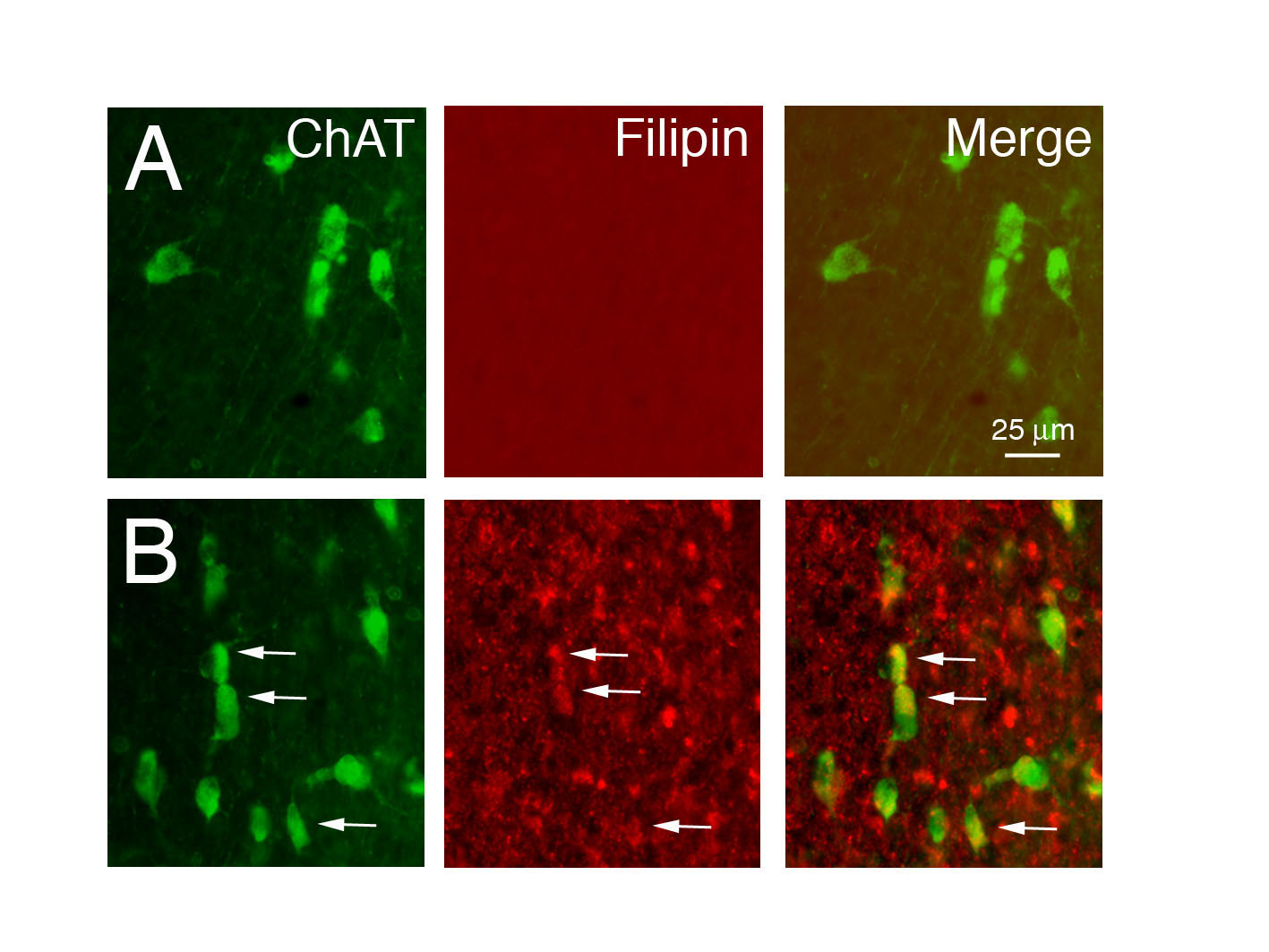

Supplement: Additional file 3 — Figure S3. Cholesterol overload in septal cholinergic neurons of NPC1-/- mice. Brain sections from 8-week-old WT mice, shown in A, and NPC1-/- mice, shown in B, were double-labeled with a polyclonal antibody against ChAT (labeling septal cholinergic cells, shown in green) and filipin (labeling cholesterol, shown in red). The arrows in B indicate cholinergic neurons that clearly demonstrate an accumulation of cholesterol in the soma. [file 1750-1326-7-11-S3.JPEG]

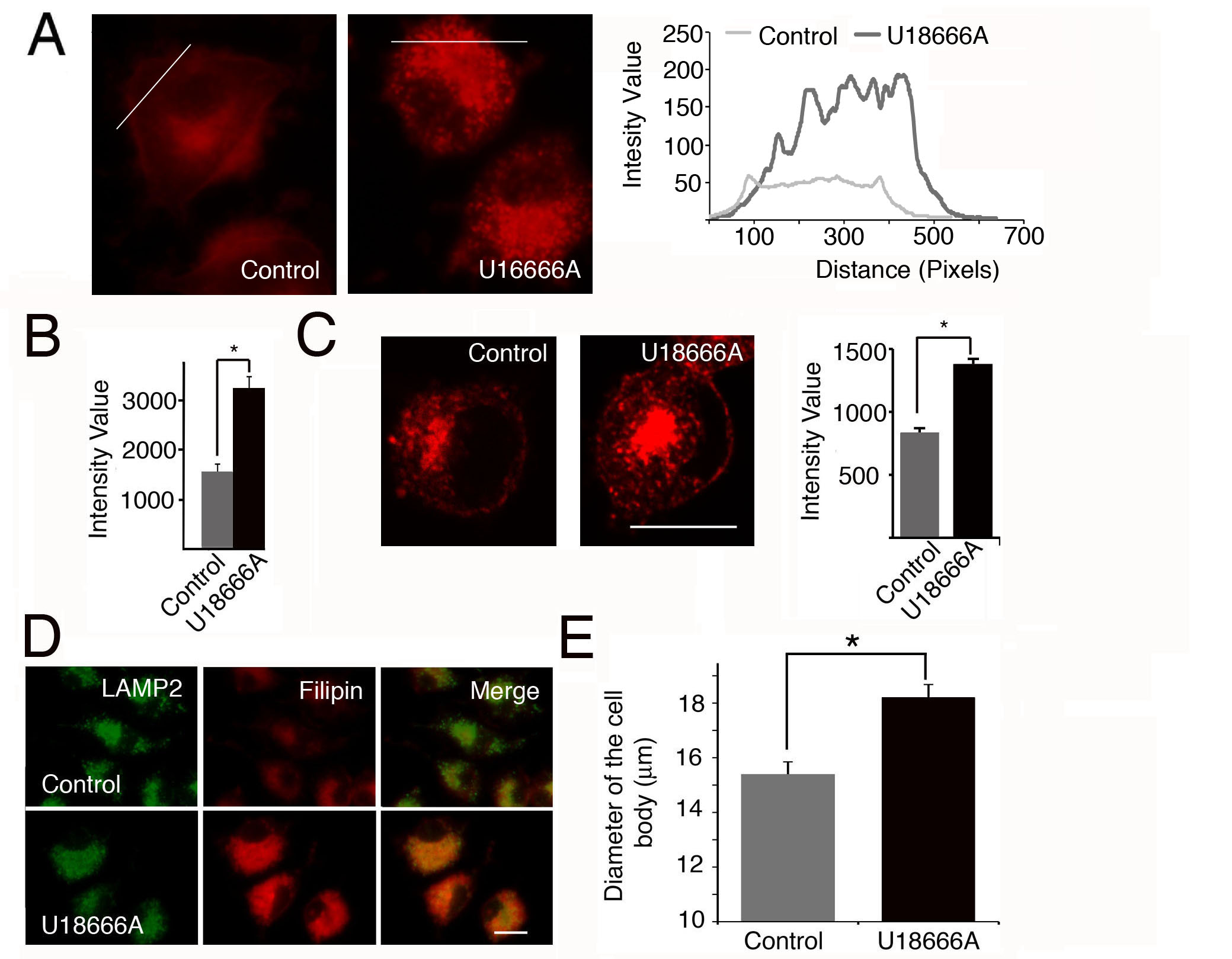

Supplement: Additional file 5 — Figure S5. PC12-U18666A cells have an NPC-like phenotype. Accumulation of cholesterol and GM1 ganglioside. A. PC12 cells were not treated (control) or treated with (2 μg/ml) U18666A for 24 hrs and stained with filipin (shown in red) before fixing with paraformaldehyde. Left panels, The fluorescence intensity profile of the red line drawn in the control and PC12-U18666A cells is shown. This result indicates that there is a difference in the distribution of cholesterol-labeled organelles. B. Quantification of total fluorescence intensity of filipin staining in control and U18666A-treated (2 μg/ml) PC12 cells. These results indicate that PC12-U18666A cells exhibit increased intracellular accumulation of cholesterol. C. Control or U1866A-treated PC12 cells were incubated with Alexa Fluor 555-conjugated cholera toxin subunit-B (CT-B) (20 μg/mL) for 30 minutes to label the accumulation of GM1 gangliosides in endosomes. The images were acquired under a confocal microscope. Left panel, There is a significant increase in the accumulation of GM1 in PC12-U18666A cells compared to control PC12 cells, *p < 0.0001, unpaired Student's t-test. n = 470-490 cells per treatment from two different experiments. D. PC12 cells were treated with 2 μg/ml U18666A for 24 hrs before staining with filipin (shown in red), fixing with paraformaldehyde and immunostaining for the lysosomal marker LAMP2 (shown in green). LAMP2 immunostaining indicates changes in the distribution of lysosomes from a perinuclear to a widespread distribution similar to the cholesterol-labeled organelles shown in A. E. The graph indicates an increase in the diameter of PC12-U18666A cells compared to control PC12 cells, p < 0.0001. [file 1750-1326-7-11-S5.JPEG]

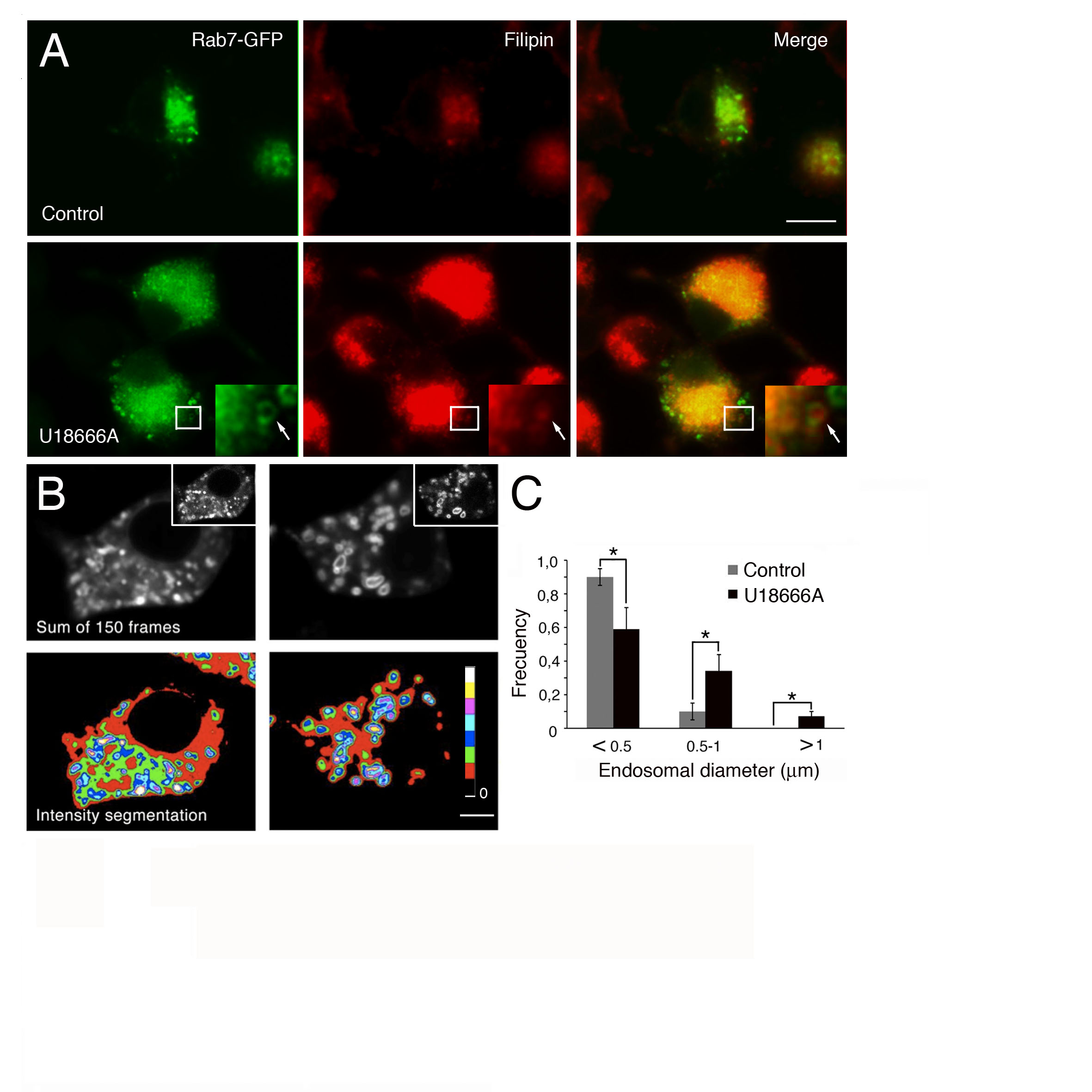

Supplement: Additional file 6 — Figure S6. Rab7-positive endosomes are abnormal in PC12-U18666A cells. A. PC12 cells were transiently transfected with Rab7-EGFP (late endosome marker, shown in green) and, after 24 hrs, treated with 2 μg/ml U18666A (U18666A) for another 24 hrs before filipin staining (shown in red). The white arrow indicates a giant Rab7 endosome overloaded with cholesterol. It is also evident that the distribution of Rab7 vesicles (control) changed as a result of treatment with U18666A. B. PC12 cells were transfected with a Rab7-EGFP plasmid and treated with NGF for 24 hrs in the presence (U18666A) or absence (control) of U18666A, and the dynamics of Rab7-positive vesicles were compared. The movement of Rab7-positive vesicles was studied by confocal microscopy of living cells with a frequency of 100 frames/min. The first frames of the sequences for the control and PC12-U18666A cells are shown as insets in the upper panels. Then, we condensed 150 frames to 1 frame, shown in the upper panel. High levels of fluorescence in the projections are a consequence of the presence of static particles. In the lower panel, the same projections are shown but with segmentation of intensity ranges into a pseudo-colored scale. Sites with static endosomes are pink or yellow, and sites with more dynamic changes in intensity are green. Note that green areas are less apparent in PC12-U18666A cells, indicating that most of the Rab7-positive endosomes are static. C. Endosomes of five control and five PC12-U18666A cells were measured and categorized into three size ranges (smaller than 0.5 μm, between 0.5 and 1 μm and bigger than 1 μm). The plot shows a histogram of the endosome size distribution in control or PC12-U18666A cells. The relative abundance (frequency) of smaller endosomes is significantly decreased in U18666A-treated cells, while medium-sized endosomes are more abundant. PC12-U18666a cells also contain enlarged endosomes. The differences between treatments are significant, *p < 0.05. [file 1750-1326-7-11-S6.JPEG]

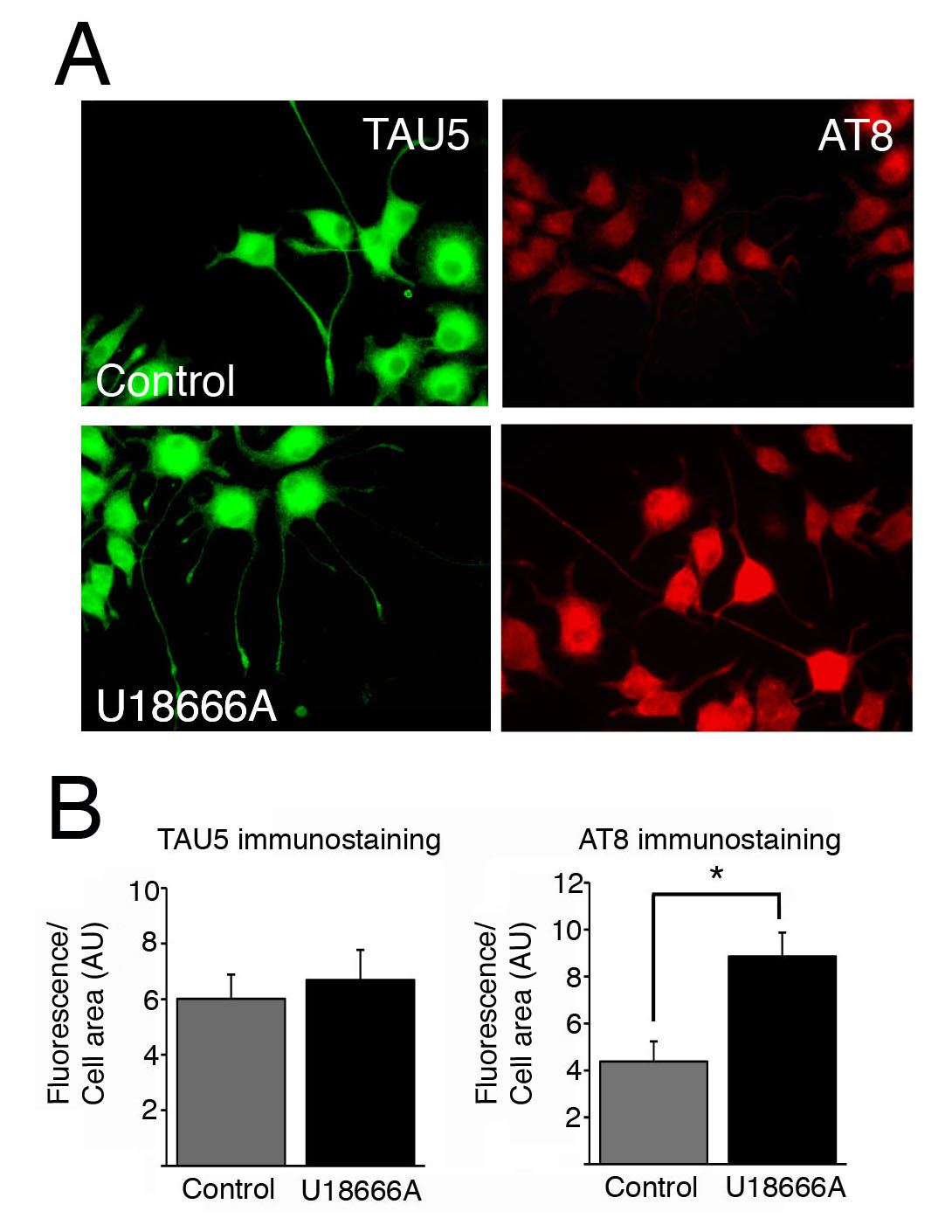

Supplement: Additional file 7 — Figure S7. Increased AT8 immunolabeling in differentiated PC12-U18666A cells compared to controls. A. PC12 cells were treated for 24 hrs with 2 μg/ml U18666A and for another 48 hrs with the same dose of the drug in the presence of NGF (5 ng/ml). After the treatments, PC12 cells were fixed and immunostained with the TAU5 monoclonal antibody, which labels total tau (green), or the AT8 monoclonal antibody against phosphorylated tau (red). PC12-U18666A cells have increased size and increased immunoreactivity against phosphorylated tau in the cell body and neurites. B. The intensity of the immunostaining of PC12 cells labeled with the TAU5 (which labels nonphosphorylated epitope in Tau) or AT8 antibody was quantified in the cell bodies of approximately 50 cells. The total fluorescence level of each cell was divided by the total area of the cell. The experiment was repeated three times with the same result, and one representative experiment was quantified. There was no significant difference in the fluorescence levels of TAU5 immunolabeling in control and PC12-U18666A cells, p = 0.6245. The differences in fluorescence levels of AT8-immunolabeling in control and PC12-U18666A cells are significant, *p < 0.005. [file 1750-1326-7-11-S7.JPEG]
